# Miniaturized Micrometer-Level Copper Wiring and Electrodes Based on Reverse-Offset Printing for Flexible Circuits

**DOI:** 10.1021/acsaelm.5c00230

**Published:** 2025-04-02

**Authors:** Kim Eiroma, Asko Sneck, Olli Halonen, Tuomas Happonen, Henrik Sandberg, Jaakko Leppäniemi

**Affiliations:** † VTT Technical Research Centre of Finland, Ltd., Tietotie 3, FI-02150 Espoo, Finland; ‡ VTT Technical Research Centre of Finland, Ltd., Kaitoväylä 1, FI-90590 Oulu, Finland

**Keywords:** high-resolution printing, reverse-offset printing, copper wiring, intense pulsed light sintering, low-temperature process, flexible circuits, component assembly, sustainable electronics

## Abstract

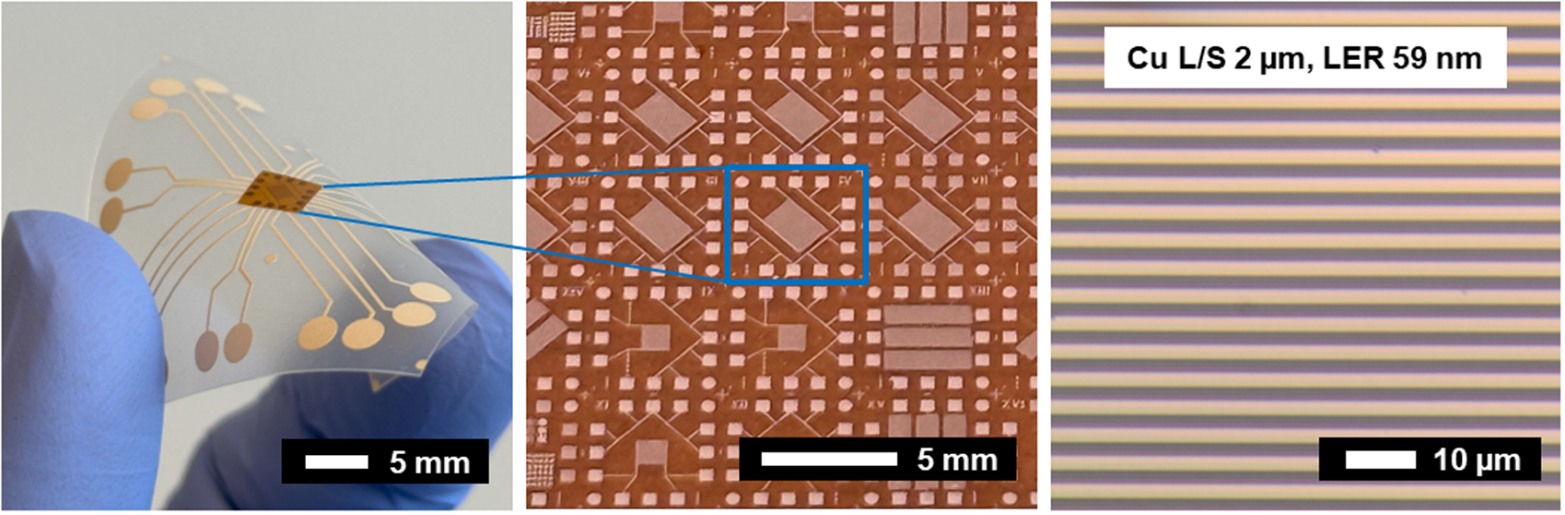

High-resolution reverse-offset printing (ROP) is developed for miniaturization of printed electronics, resulting in a notable decrease in material usage compared to conventional printing processes. Two alternative ROP processes for patterning of metal conductors are available that are comparable in their cost per sample: direct nanoparticle (NP) printing (e.g., Ag and Cu) and patterning of vacuum-deposited metal (Ag, Al, Au, Cu, Ti, etc.) films using ROP printed polymer resist ink and the lift-off (LO) process. In this work, we focus on ROP of Cu NP ink followed by intense pulsed light (IPL) sintering and vacuum-deposited Cu patterned by ROP lift-off (LO). The good large-scale uniformity of the two processes is demonstrated by a grid of 300 individual thickness, sheet resistance, and resistivity measurement points with low variation over the 10 cm × 10 cm printed sample area. Sheet resistances of 0.56 ± 0.03 and 1.23 ± 0.05 Ω/□ are obtained at 113 and 40 nm thickness for Cu NP and Cu LO, respectively. Both processes show <5% thickness variation over a large area. A line-space (L/S) resolution of 2 μm is obtained for ROP patterned vacuum-deposited Cu having very low line edge roughness (LER) (∼60 nm), whereas for direct ROP printed Cu NP ink, the L/S resolution (2–4 μm) is limited by LER (∼900 nm) and influenced by the printed layer thickness. Based on the two fabrication routes, a flexible chip component assembly process is presented. Preliminary bending resistance results indicate that both ROP-based patterning processes yield a robust electrical interconnection between the ultrathin polyimide (PI) 5 mm × 5 mm chip and thermoplastic polyurethane (TPU). ROP shows promise as a scalable and sustainable patterning method for flexible ICs/chips that are assembled on flexible, stretchable, or biodegradable substrates and used, e.g., in wearable, large-scale sensing, and in environmental monitoring.

## Introduction

Printing is generally regarded as a sustainable alternative manufacturing method to the conventional photolithography-based fabrication of electronics and is especially well-suited for wearable and large-scale sensing applications. Many printing methods can be considered material conservative and additive when compared to the subtractive processes of etching and lift-off that are used in conventional electronics manufacturing.^1^ Although some factors impacting on the material usage like the excess ink needed in the printing process are acknowledged,^1^ the amount of material in the final product is often overlooked in discussion. For example, conventional printing methods, such as inkjet printing and screen printing, produce wide lines above 30 μm line widths and film thickness in the micrometer range. This leads to substantial material volume in the final product when compared to the submicrometer line widths and thicknesses obtained with conventional lithography processes. Although some applications such as antennas require thick films to avoid resistive capacitive (RC) delay, resistive losses, and to provide sufficient skin depths for different antenna frequencies,^2^ many applications like active components (thin-film transistors, diodes, memristors) and sensors would benefit from thin films and narrow line widths (higher device density and smaller circuit footprint).^3^ Novel printing methods such as electrohydrodynamic (EHD) jet, super inkjet (SIJ), ultraprecise dispensing (UPD), and reverse-offset printing (ROP) have been developed to allow miniaturization of printed features, down to single micrometer-level feature sizes.^4−7^ These methods can produce conductive structures at a fraction of the material usage compared to conventional printing methods. Nozzle-based methods like EHD, SIJ, and UPD have a distinct advantage in that they can be utilized to pattern materials on three-dimensional (3D) surfaces and/or to build 3D structures using the printed material.^8^ However, scaling up of the aforementioned nozzle-based methods to large areas and/or dense patterning poses challenges, whereas a large area defined by the size of the printing plate (cliché) can be patterned by ROP in a single pass, enabling manufacturing throughput and resolution on a competitive level compared to conventional microelectronics processes. Also, the ability to pattern thin layers with nearly rectangular side profiles that are closely matching the intended pattern layout design dimensions is a unique benefit of ROP for fabricating multilayer devices.

Another aspect impacting the sustainability of electronics manufacturing is the choice of conductive materials. Silver (Ag) is the most applied conductive nanoparticle (NP) ink thanks to its low resistivity (ρ) of 1.59 μΩ·cm, simple and low-temperature sintering process, and good oxidation stability along with a conductive oxide phase.^9,10^ However, Ag suffers from high cost, low electromigration resistance, and environmental concerns. Copper (Cu) is considered a more abundant, low-cost, stable, and sustainable alternative to Ag that provides a low ρ of 1.72 μΩ·cm, thus close to Ag.^9,11^ However, Cu requires special attention in the sintering process as Cu NPs readily oxidize and copper oxides are nonconductive. Several methods have been developed for sintering of Cu NP inks that overcome oxidation: thermal sintering in reductive (forming gas, vacuum, or oxygen pump) or inert (N_2_) atmosphere, or rapid sintering via laser, or intense pulsed light (IPL).^12−17^ Especially, the IPL process allows treatment of large areas concurrently and avoids excessive heating of the substrate, thus enabling the use of flexible substrates with lower thermal tolerance than glass.^18^ In addition to Cu, other alternatives, such as aluminum (Al) and carbon (C), could provide an even lower cost and more sustainable approach than Cu but suffer from rapid oxidation as NPs and high ρ, respectively.^9^

Table S1 summarizes some key prior work on Cu conductors using different printing methods. Clearly, the high-resolution printing of Cu conductors would bring sustainability advantages in terms of material conservation as several orders of magnitude less material is needed to produce one square of Cu conductor using higher-resolution printing methods than screen or inkjet printing. Earlier, printing of miniaturized Cu wiring and electrodes with line width resolution ≤10 μm has been demonstrated by combining SIJ and oxygen pump sintering of Cu NPs (5 μm line width), ROP and IPL sintering of Cu NPs (10 μm line width), and ROP and IPL sintering of Cu NWs (7 μm line width).^14,19,20^ However, no demonstrations below 5 μm line widths have been published so far. In this paper, we show that Cu wiring and electrodes can be patterned using ROP down to 1–2 μm line widths on flexible substrates, namely, polyimide (PI) and polyethylene naphthalate (PEN), using two different ROP-based methods: (i) combining ROP of Cu NPs and IPL and (ii) combining ROP of polymer resist and lift-off (LO) process of vacuum evaporation of Cu.^21^ Both ROP-based Cu electrode patterning methods yield low resistivity (ρ) of 6.3 and 4.9 μΩ·cm, and sufficient sheet resistance (*R*_s_) of 0.56 and 1.23 Ω/□ at ∼113 and 40 nm thickness (*t*) for Cu NPs and Cu LO process, respectively. Here, we also show that the ROP LO process can be extended to high-resolution (1–4 μm) patterning of various metal electrodes including Al, Ag, and gold (Au) on both PI and PEN substrates. Using the thickness values and the sample dimensions from this work, the materials consumption and cost for the two processes as well as factors affecting the environmental impact are analyzed in Figure S1 and Table S2, which indicate that both processes are comparable in terms of materials cost per unit area. Notably, the cost for the NP process for fixed thickness is highly dependent on the cost of the nanoparticle ink (will scale down at bulk volumes), the weight percentage (wt %) of NPs in the ink, and the density of the final sintered layer. For the ROP LO process, the cost is mostly affected by the lift-off solvent consumption (solvent price, usage per sample, and recycling/reusing of the solvent). Moreover, we systematically study uniformity of the *t*, *R*_s_, and ρ for both ROP-based Cu patterning processes over a 10 cm × 10 cm sample area at 300 measurement points. Finally, to simulate the use of ROP-based flexible chips in wearable applications, we fabricated 5 mm × 5 mm test chips on ultrathin 38-μm-thick PI and 125-μm-thick PEN and successfully assembled those onto stretchable thermoplastic polyurethane (TPU) substrate using an electrically conductive adhesive. Both ROP-based Cu methods demonstrated sufficient Cu layer thickness for the flexible, ultrathin PI chip assembly process, although Cu NPs show potentially better robustness in initial bending tests thanks to their higher layer thickness. The results demonstrate the large-area reproducibility (yields > 99% and parameter variations at 4–6% level), scalability (400 pieces of 5 mm × 5 mm sized chips per single print), and good applicability (integration to larger stretchable substrates) of both ROP-based high-resolution Cu patterning processes.

## Experimental Section

A sheet-fed reverse-offset printing system (Jemflex/Nihon Denshi Seiki) was used to print a Cu NP ink (CUNI-AC-01, Ishihara Chemical Co., LTD) and a poly-4-vinylphenol (PVPh)-based polymer resist ink on a 38-μm-thick PI substrate (Xenomax, Nagase) and a 125-μm-thick PEN substrate (Teonex Q 65HA, DuPont Teijin Films). An Ag NP ROP ink (L-Ag RPO, Ulvac) was printed on the PI substrate as a reference. The commercial Cu NP ink is optimized for adhesion contrast planography (ACP), a poly(dimethylsiloxane) (PDMS)-based printing process, and intense pulsed light sintering (IPL).^22^ The polymer resist ink developed for patterning of vacuum-deposited materials was prepared by dissolving PVPh into ethyl lactate and ethyl acetate solvents at 3.5 wt % solids loading.^21^ A polymer surface additive (BYK-355) was added to enhance the leveling and anticratering properties of the ink. The substrates were attached on a 125 mm × 125 mm glass carrier substrate using a cool-off-type laminating tape (Plafix, NITTA) in order to facilitate the subsequent dicing of the substrate into flexible IC test chips for component assembly tests. The high-resolution printing cliché was fabricated on a silicon wafer using photolithography and deep reactive ion etching.^21^

The printing system is located in a semiclean room environment with controlled temperature and humidity. The ROP process was initiated by coating a thin and uniform layer of ink onto a PDMS blanket surface. The inks were conditioned prior to use by shaking (>30 min) and the NP inks were additionally treated in an ultrasonic bath (∼5 min). The surface energy of the PDMS surface was increased by low-pressure O_2_ plasma (13.56 MHz, 60 W, 30 s) (Nano, Diener Electronic) to enhance the wetting of the polymer resist ink. The Cu NP ink did not require plasma treatment of the PDMS. The coating was performed using a glass slit capillary coater into which an optimized volume of ink (300 μL) was pipetted manually. After the coating step, the ink layer was allowed to reach a semidry state through partial absorption of the solvents into the PDMS and through evaporation. In the OFF step, the relief pattern of the printing cliché was brought into contact with the semidry ink film on the PDMS roller surface. A minimal indentation pressure was applied in the printing nip, and the relief pattern edges fractured the semidry ink film, and the areas that met the raised portions of the relief pattern were removed by the cliché. In the SET step, the pattern remaining on the PDMS roller surface was transferred to the substrate by applying a minimum indentation pressure in the PDMS roller–substrate nip. The transfer of the semidry ink layer from PDMS to cliché in the OFF step and from PDMS to substrate in the SET step occurs when the prerequisites of sufficient cohesion of the ink layer and of higher adhesion of the ink layer to cliché and substrate than to the PDMS are met.^23^ The substrate surface was treated by low-pressure O_2_ plasma in order to ensure good transfer and adhesion of the semidry ink layer to the substrate.

Two ROP-based processes were used to pattern the metals. For direct ROP printing, a Cu NP ink was used. Two coating speeds were used for fabricating samples with different layer thicknesses, 5 mm/s for thin layers (lt) and 10 mm/s for thick layers (ht), and for the OFF and SET steps, the process speed was 20 mm/s. The ink was dried on a hot plate immediately after printing for 1 min at 80 °C. The samples were stored in a nitrogen-filled glovebox prior to IPL sintering that was performed using a broad band spectrum xenon lamp system (Xenon Sinteron S-2100, Xenon Corp.) with a type B lamp having a spectral cutoff at 240 nm. The IPL sintering parameters were optimized by varying the pulse voltage amplitude (1800–3000 V) and width (250–2000 μs) in a parameter matrix (Tables S3 and S4). The optimized 3 kV/1250 μs pulse gives a calculated total energy dose of 1138 J per pulse for the PI substrate. These parameters yielded the lowest resistance without visible adverse effects such as ablation of the Cu NP material or deformation or melting of the substrate. Conversely, for the PEN substrate, a parameter set could not be found for successful sintering of Cu NP material without ablation or substrate melting. The 10 cm × 10 cm printed sample was transported at 5 mm/s under the 19 mm × 305 mm xenon lamp, and a single pulse was flashed every 5 mm with the lamp width perpendicular to the printing direction, ensuring ample overlap between treated areas. The reference Ag NP ink was oven-sintered in air at 150 °C for 30 min. For indirect metal patterning using the ROP LO process, a negative of the intended pattern was printed using the polymer resist ink and the resulting polymer had sharp vertical sidewalls that are ideal for the LO process.^21^ The coating speed was 10 mm/s, and for the OFF and SET steps, the process speed was 20 mm/s. Subsequently, 40 nm of metal was deposited via electron-beam evaporation (MinilabET080A, Moorfield Nanotechnology). The e-beam was centered under a rotating sample at a distance of 55 cm. Finally, the underlying resist layer was removed in a lift-off process using deionized water/ethanol or methanol in a Megasonic bath (Sonosys Ultraschallsysteme), resulting in the positive metal pattern remaining on the substrate. Metals patterned following the ROP LO process were Cu, Au with a 5 nm titanium adhesion layer, Ag, and Al.

The dimensional and morphological quality of the printed samples was characterized by optical microscopy (BX50, Olympus), stylus profilometry (Dektak 150, Veeco), scanning electron microscopy (SEM) (Supra 35, Zeiss), and atomic force microscopy (AFM) (Dimension 3100 SPM, Digital Instruments). Electrical characterization was performed using a 4-terminal resistance measurement setup (Keithley 2700 Multimeter). The adhesion of the ROP patterned Cu layers on the PI substrate was evaluated by adapting the ASTM D 3359-02 Standard Test Method.

**Figure 1:**
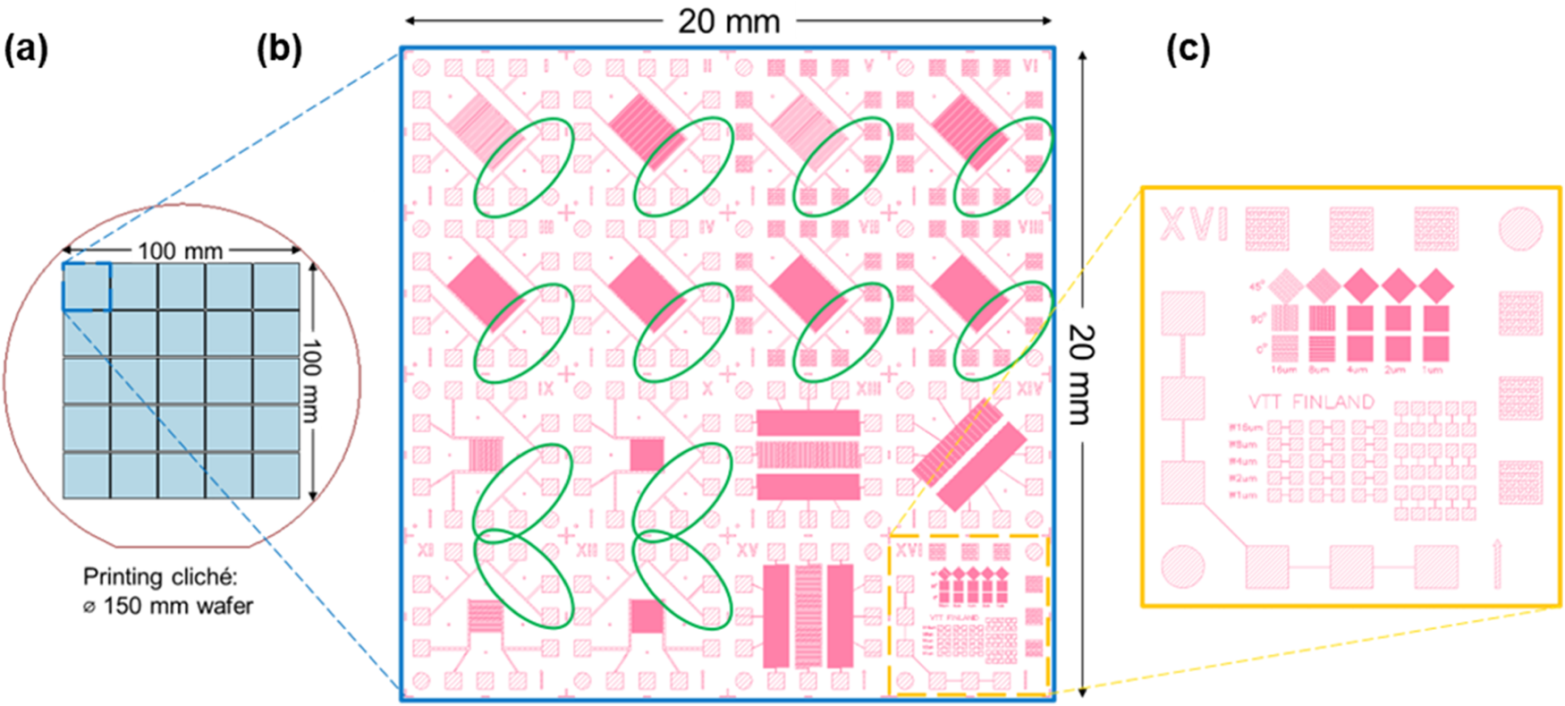
20 mm × 20 mm printing pattern (b) repeats over a 100 mm × 100 mm area on printing cliché (a). Each pattern consists of 16 patterns of 5 mm × 5 mm, of which 12 are intended for flexible IC assembly tests. Large-area uniformity measurements were performed on the conductor structures (*W* = 50 μm, *L* = 1000 μm) indicated in green. In subsequent assembly tests, the 5 mm × 5 mm patterns were separated using a dicing saw and assembled onto a stretchable TPU substrate using a conductive adhesive for characterization of the interconnection. Basic morphological and electrical characterization was performed using test structures indicated in yellow (c).

The print pattern and cliché layout are presented in Figure 1. The 10 cm × 10 cm printing area consisted of 25 repeating 2 cm × 2 cm print patterns (Figure 1a). Each print pattern (Figure 1b) consists of 16 areas of 5 mm × 5 mm, which represent a flexible dummy test chip with interconnected 500 μm × 500 μm contact pads at edges for assembly tests on stretchable TPU. The top right quadrant of the layout contains 4 chips with contact pads having a patterned mesh structure with a line width and hole dimension of 45 μm. The lower right 5 mm × 5 mm pattern, marked in yellow, contains test structures for basic morphological and electrical characterization of all fabricated samples (Figure 1c). In addition, the uniformity was characterized from the Cu NP direct ROP and Cu ROP LO samples over the entire 10 cm × 10 cm sample area. The measured locations (12 out of 16) are marked in green (Figure 1b). The bottom right quadrant of the layout was dedicated to a set of other test patterns without interconnected contact pad patterns. In total, 300 locations were characterized for their resistance and thickness profiles using stylus profilometry.

The samples were diced into 5 mm × 5 mm chips for the flexible IC component assembly test. The individual chips were then delaminated from the interliner whose bonding strength weakens after storing them in a refrigerator. The samples were placed on a component carrier tape. Isotropic conductive silver adhesive (ICA) (EPO-TEK H20E-PFC) was dispensed onto a screen-printed Ag electrode pattern on a stretchable 100-μm-thick TPU substrate (Platilon U073, Covestro) attached to a 75-μm-thick polyethylene carrier film, the flexible IC test chip was picked up from the carrier tape, aligned, and placed onto the Ag electrodes, and finally dried at 120 °C for 15 min to cure the ICA.^24^ After assembly of the flexible IC test chips on TPU, the mechanical robustness and electrical resistance of the interconnection was evaluated by conducting a manual bending test over three different bending radii (28, 15, and 8 mm). The 4-point resistance between two interconnected contact pads on the flexible IC chip was measured via the screen-printed Ag electrode.

## Results and Discussion

**Figure 2:**
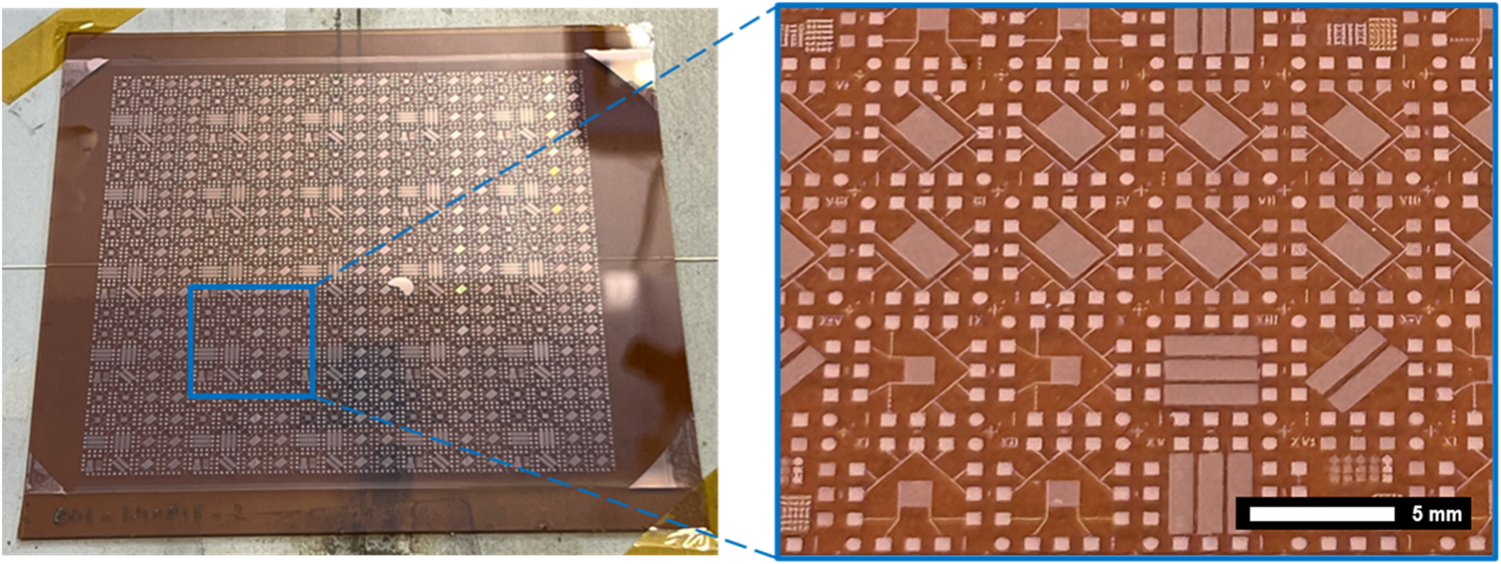
10 cm × 10 cm Cu NP on PI substrate after the IPL sintering process.

Cu NP samples on the PI substrate and Cu LO samples on both PI and PEN substrate were successfully fabricated. Figure 2 shows a 10 cm × 10 cm Cu NP sample on the PI substrate after IPL sintering using the optimized parameters, where a shiny metal color could be observed after the IPL (Figure S2). ROP LO was also successfully demonstrated for patterning of other metals: Ag, Au, and Al. As ROP LO is a low-temperature process, patterning on a low glass transition temperature (*T*_g_) substrate, such as PEN, is possible. Cu NP samples on PEN were successfully printed with ROP. However, even though IPL sintering of Cu oxide NP and Ag NP on low *T*_g_ polyethylene terephthalate (PET) substrate has been demonstrated in a prior work,^25^ the optimization of IPL sintering parameters for the Cu NP ink was not successful on PEN. A very narrow operating window was observed where initially the melting of the substrate occurs with occasional kΩ–MΩ level resistance measured, followed by partial ablation of the Cu NP layers as the sintering energy is increased (Figure S3). The IPL parameter optimization matrix is presented in Tables S3 and S4 for the PI and PEN substrate, respectively, together with qualitative observations for each parameter combination.

**Figure 3:**
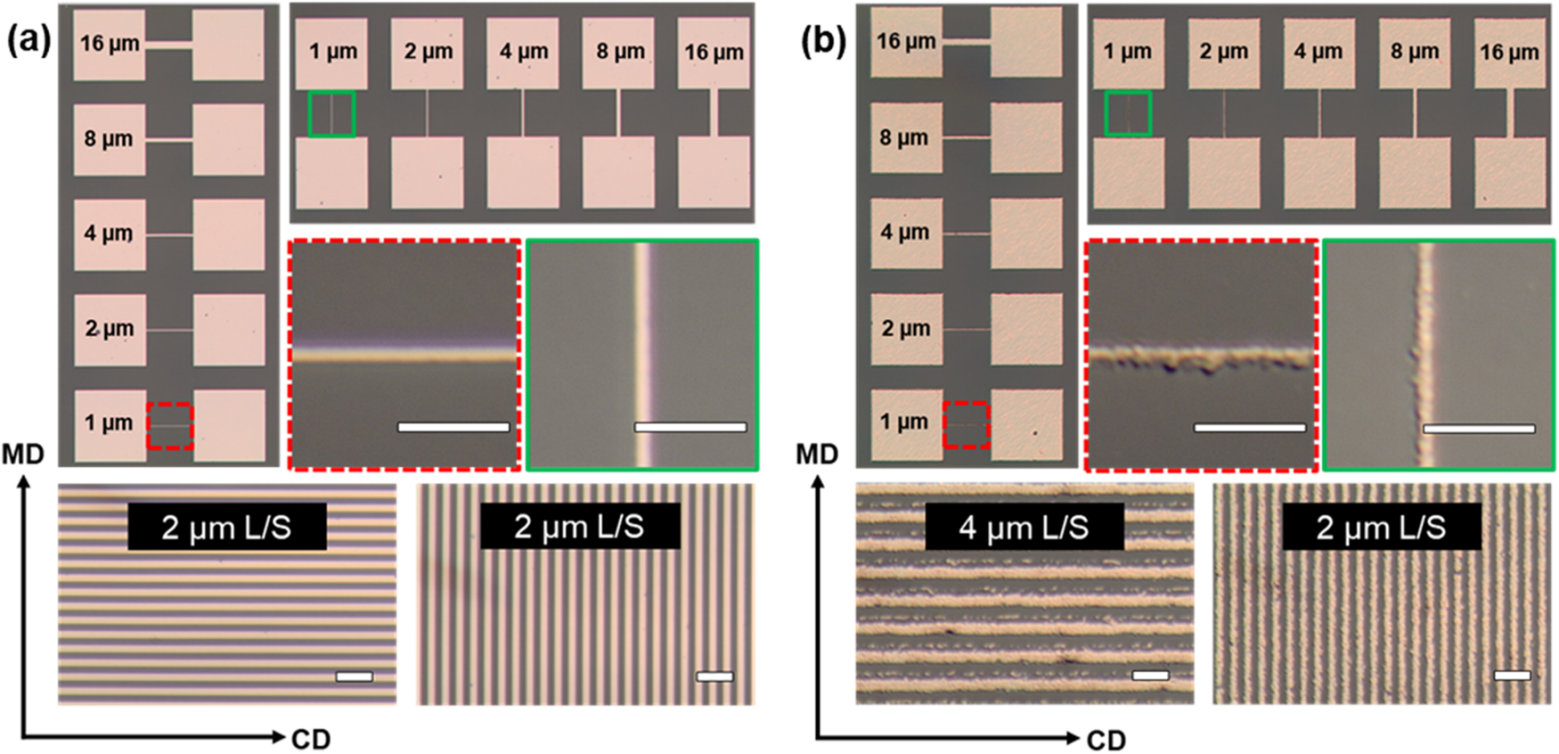
Print-quality comparison between Cu LO (a) and Cu NP (lt) (b) patterns on a PI substrate. The minimum achieved L/S patterning (*W*_min_ (L/S)) is shown in MD and CD. The minimum achieved isolated line width (*W*_min_ (line)) is indicated in green (MD) and red (CD). Scale bar: 10 μm.

Print quality and electrical characterization data for all fabricated samples are summarized in Table 1. The print quality is evaluated by the minimum isolated line width (*W*_min_ (line)) for a continuous, isolated line and the minimum achievable L/S resolution (*W*_min_ (L/S)) for both machine (MD) and cross-machine direction (CD). For Cu NP direct ROP, Cu NP (ht) and Cu NP (lt) indicate the fabricated high (∼127 nm) and low (∼101 nm) layer thickness (*t*) samples, respectively. The results demonstrate that the highest patterning resolution is achievable with thinner layers. This is in line with prior work suggesting a larger printing parameter window for thinner ink layers.^23,26^*W*_min_ (line) is 1 μm for Cu NP (lt) and all metals patterned by ROP LO. The reference Ag NP ink (*t* ∼ 225 nm) yields 2 μm *W*_min_ (L/S) and 2 μm *W*_min_ (line) on the PI substrate in both MD and CD. Figure 3 shows a comparison of Cu LO and Cu NP (lt) printing quality characteristics on the PI substrate. For ROP LO, the polymer resist patterning resolution is likewise determined by the thickness of the resist ink layer in the coating step of the ROP process. The thickness of the deposited metal and subsequent metal patterning by LO is optimized relative to the resist thickness. In general, the resist layer thickness should be at least 1.2−1.3× of the metal thickness for successful LO patterning.^27^ The polymer resist ink used in this work is optimized for ROP of ∼70-nm-thick polymer layers, which guarantees good-quality patterning for metal thickness of ∼40 nm used in this work.^28^ 40 nm is sufficiently thick to yield close to bulk material resistivity and to avoid a thickness-dependent resistivity increase, as has been observed, e.g., for ultrathin <40 nm Cu layers.^29^

**Table 1:** Basic Print Quality and Electrical Characterization Data for Direct ROP Cu NP and ROP LO Patterned Metal Layers

	print quality				electrical characterization			
material/substrate	*W*_min_ (MD/CD)^b^		*t*	*W*^c^	*R*^c^	*R*_s_	ρ	ρ
	μm (line)	μm (L/S)	nm	μm	Ω	Ω/□	μΩ·cm	*x* bulk
Cu NP (ht)^a^/PI	2/4	4/4	127	13.5	5.38 ± 0.20	0.73 ± 0.03	9.23 ± 0.35	5.43
Cu NP (lt)^d^/PI	1/1	2/4	101	15.5	6.94 ± 0.20	1.07 ± 0.03	10.88 ± 0.31	6.40
Cu LO/PI	1/1	2/2	39	15.5	8.84 ± 0.27	1.37 ± 0.04	5.33 ± 0.16	3.14
Cu LO/PEN	1/1	2/2	39	15.5	10.37 ± 0.26	1.61 ± 0.04	6.25 ± 0.16	3.68
Ag LO/PI	1/1	2/2	38	15.6	7.52 ± 0.11	1.17 ± 0.02	4.44 ± 0.07	2.78
Ag LO/PEN	1/1	4/4	38	15.6	7.19 ± 0.11	1.12 ± 0.02	4.24 ± 0.07	2.65
Au LO/PI	1/1	2/2	44	15.7	11.76 ± 0.26	1.84 ± 0.04	8.04 ± 0.18	3.65
Au LO/PEN	1/1	2/2	44	15.7	11.10 ± 0.14	1.74 ± 0.02	7.59 ± 0.10	3.45
Al LO/PI	1/1	4/4	44	15.5	8.40 ± 0.17	1.30 ± 0.03	5.66 ± 0.11	2.18
Al LO/PEN	1/1	4/4	39	15.5	7.96 ± 0.34	1.24 ± 0.05	4.81 ± 0.20	1.85

a ht = high thickness.

b Nominal line width.

c Nominal W/L = 16/100 μm.

d lt = low thickness.

The results for the measured line widths (*W*) show that for both ROP Cu NP and ROP LO processes, the printed *W* is very close to the nominal 16 μm width (variation ≤ 0.5 μm) when measured from the line profile full width at half-maximum (fwhm) value. A minimal deviation from nominal dimensions is typical for ROP, due to pattern profiles exhibiting close to vertical sidewalls as a result of patterning of the ink layer that is semidry and has sufficient cohesion to overcome the adhesive forces present between the ink layer and the PDMS surface in the OFF step.^23^ A notable exception is observed for Cu NP (ht) (13.5 μm), which shows a significant deviation from the nominal line width (16 μm). Cu NP (ht) and Cu NP (lt) are compared side by side in optical micrographs in Figure S4. The leading edge of the CD lines in the 4 μm L/S patterns shows a likely artifact of imperfect patterning in the OFF step that is more pronounced for the Cu NP (ht) sample with a higher thickness. An overall rougher line edge in MD and CD is also observed for Cu NP (ht) compared to Cu NP (lt). A key contributing factor to the patterning quality in PDMS-based patterning methods, such as ROP and ACP, is the degree of the semidry state, i.e., the volume fraction (φ) of the Cu NP to residual solvent in the semidry ink layer. If φ is too low, the layer will not fracture under strain ideally like an elastic solid-like film, but the response is more viscous-dominant. This leads to sloped sidewalls and poor edge definition, but notably the widening of lines, which is contrary to our observation.^30^ The deviation from nominal *W* for Cu NP (ht) and the observed notable LER could be the result of the region immediately outside of the patterning area (cliché edge) separating due to insufficient adhesion of the semidry ink to the PDMS. While the cohesion of the semidry ink is initially large enough to resist the fracture in the nip of the OFF step, the fracture eventually occurs at a less defined location close to the pattern edge as the cliché is removed. The length of the separated region (skirt) and the location at which the skirt partially breaks increases with increasing semidry layer thickness.^31^ This would be manifested as a notable LER, which seems to be in agreement with our observations for Cu NP (ht) and Cu NP (lt). The process could be optimized by further reducing the semidry layer thickness (at the expense of *R*_s_) and optimizing the balance between cohesion and the adhesion of the semidry ink layer to the PDMS surface by either tuning the ink composition or PDMS surface energy. However, plasma activation (e.g., O_2_) could not be used for enhancing the PDMS adhesion as it resulted in poor wetting of the Cu NP ink on the PDMS.

For metals patterned with ROP LO, a *W*_min_ (line) of 1 μm and a *W*_min_ (L/S) in the same range (2 μm at best) were obtained. For the given resist thickness of ∼70 nm, ∼2 μm L/S, and 0.5–1 μm, the isolated line resolution represents the performance limits for the ROP LO process in our experience so far.^28^ In order to go beyond <1 μm L/S resolution for ROP LO, the resist thickness would need to be thinner. The same *W*_min_ (L/S) and *W*_min_ (line) in both MD and CD for ROP LO demonstrate an important benefit of an optimized ROP process. Symmetrical micrometer-level L/S patterning, with near-vertical sidewalls made possible by patterning the ink in the semidry state, is a distinct advantage, which separates ROP from printing methods where wetting phenomena at the ink–substrate interface are difficult to control. The differences in *W*_min_ (L/S) for the different metals patterned with ROP LO may be the result of nonoptimized LO process conditions as well as adhesion of the evaporated metal. Surface roughness (nominal average roughness *R*_a,PI_ = 0.5 nm and *R*_a,PEN_ = 1.1 nm by manufacturer specifications) and conditions of the substrate may contribute to crater-like print-quality artifacts, as can be seen in optical micrographs in Figures S5–S8, even though this is not evident from *W*_min_ data in Table 1.

Overall, Cu NP (ht) and (lt) provide the lowest *R*_s_ due to a thicker layer (0.73 ± 0.03 and 1.07 ± 0.03 Ω/□, respectively), when compared to the ROP LO Cu. The thinner metals patterned with ROP LO yield consistently higher patterning resolution but exhibit higher *R*_s_ values between 1.12 and 1.84 Ω/□, depending on the metal. In comparison, a *R*_s_ of 0.63 ± 0.02 Ω/□ was obtained for Ag NP (*t* ∼ 225 nm) on the PI substrate. The metals patterned with ROP LO, expectedly, show resistivities (ρ) closer to the respective bulk metals (1.85×–3.68×), whereas Cu NP (ht) and Cu NP (lt) layers have a higher ρ compared to bulk Cu (5.43× and 6.40×, respectively).

**Figure 4:**
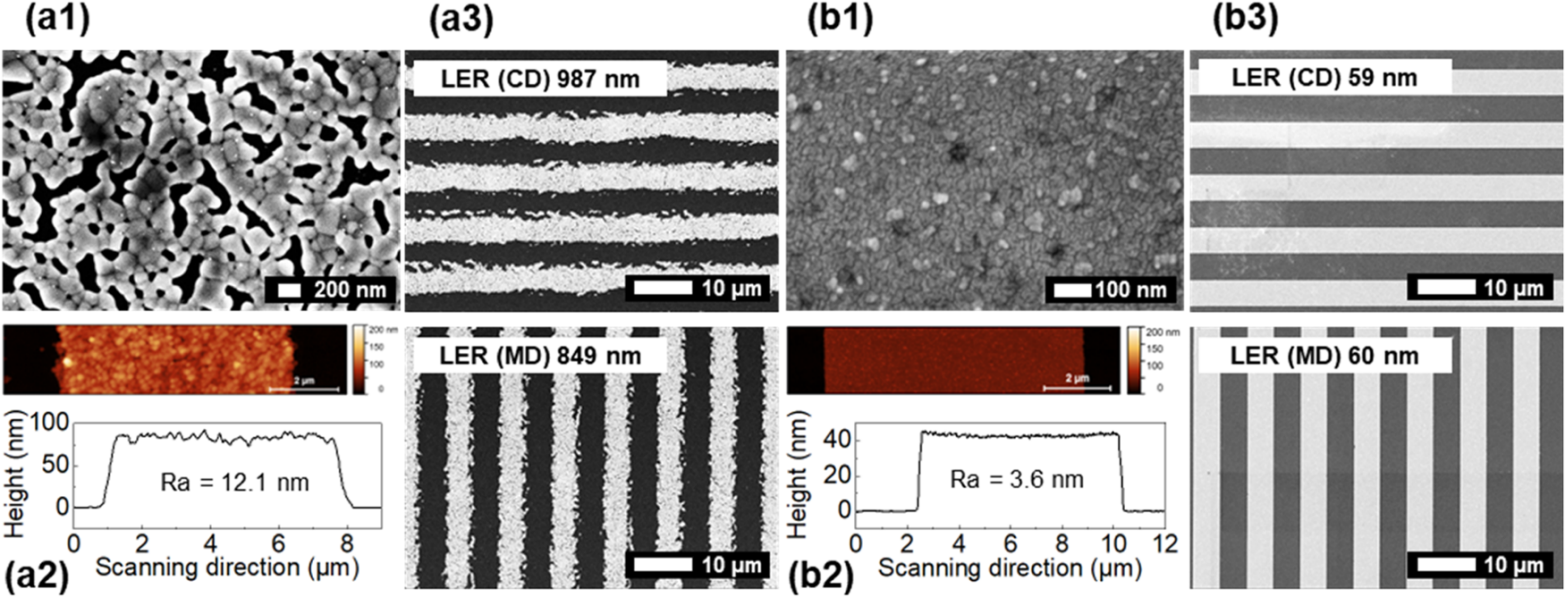
SEM images of the surface of Cu NP (lt) on the PI substrate after IPL sintering (a1) and surface of Cu LO on the PI substrate (b1). AFM scan and line profile over nominal 8 μm wide lines for Cu NP (lt)/PI (a2) and Cu LO/PI (b2). SEM images of the nominal 4 μm line-space pattern for Cu NP (lt)/PI (a3) and Cu LO/PI (b3) in MD and CD. Line edge roughness (LER) values are indicated for each case. 5 nm of Au has been deposited on the surface of the sample in order to minimize charging of the surface during imaging.

Cu NP (lt) and Cu LO on the PI substrate were compared in more detail by SEM and AFM (Figure 4). The coalescence of the nanoparticles resulted in a porous network-like morphology of the Cu NP surface after the IPL process and is evident in the SEM image (Figure 4a1), which is typical for NPs sintered with IPL.^16^ The discontinuities in the layer after coalescence of the Cu NPs are a result of the low thickness of the layer, which corresponds to the size of only a few Cu NPs. In comparison, the Cu LO surface shows a continuous finer grain morphology (Figure 4b1). The porosity of the IPL-sintered Cu NP likely contributes to the higher ρ when compared to Cu LO, as discussed above. The morphology is reflected in the measured *R*_a_ values from the AFM data for Cu NP (lt) (Figure 4a2) and Cu LO (Figure 4b2) (12.1 and 3.6 nm, respectively). The line profiles also reveal a close to vertical sidewall for the Cu LO line, whereas the sidewall is not as steep for the Cu NP (lt) line.

LER is significantly higher for Cu NP (lt) (849 nm in MD) (Figure 4a3) than for Cu LO (∼60 nm) (Figure 4b3), which is close to the typical LER (<100 nm) for ROP.^5^ Particle size and the homogeneity of the densely packed semidry nanoparticle layer have an inherent contribution to the sharpness of the fractured layer edge in the ROP patterning process. The reference Ag NP ROP ink having a particle size of 3–8 nm (according to datasheet) yielded LER values in the 120–180 nm range. For the Cu NP ink, the corresponding datasheet value for the particle size is 40 nm, which is roughly in agreement with the SEM image of the dried Cu NP (lt) layer (prior to sintering) (Figure S9). Larger clusters are, however, also present, which can originate from poor nanoparticle dispersion in the ink or be formed during the printing or drying process steps. Thus, the particle size and homogeneity can, in part, explain the higher LER values for Cu NP compared with Ag NP.

A difference in LER in MD and CD for Cu NP (lt) is also observed, with the LER value being higher in CD (987 nm). The higher LER in CD compared to MD could be explained by the difference in magnitude of the effective shear strength as the semidry layer is fractured in CD and MD. The pattern length under the printing nip is shorter in MD, and hence the effective shear strength is also smaller. For a given L/S dimension, a larger window for successful patterning is obtained with decreasing shear strength and film thickness.^23^ The high LER value for Cu NP (lt) limits the achievable L/S resolution to 2 μm in MD and 4 μm in CD. The LER of both Cu NP (ht) and Cu NP (lt) was also analyzed from optical microscope images, showing significantly higher values for Cu NP (ht) than for Cu NP (lt) (Figure S10), which further supports the mechanisms suggested above for the observed degradation of the print quality as the layer thickness increases.

**Figure 5:**
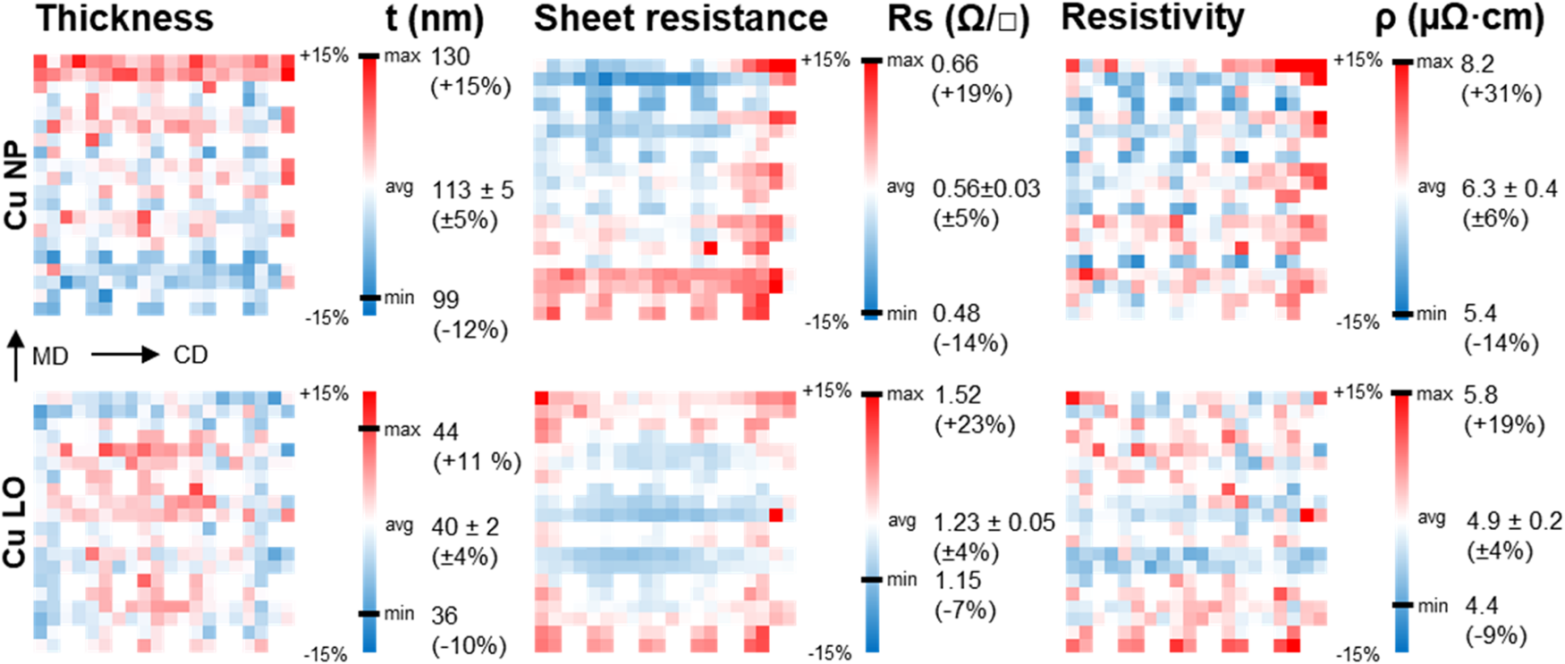
Thickness (*t*), sheet resistance (*R*_s_) and resistivity (ρ) variation, shown as a percentage deviation from the average value (white), over 10 cm × 10 cm printing area measured from (*W* = 50 μm, *L* = 1000 μm) lines at 5 mm pitch for Cu NP (ht) and Cu LO on the PI substrate. Blank 2 × 2 areas represent unmeasured locations (4/16 at the lower right within each repeating 2 cm × 2 cm print pattern). MD and CD are indicated by arrows.

The large-area uniformity of the ROP metal patterning process was studied by measuring the *t*, *R*_s_, and ρ over the entire 10 cm × 10 cm sample area at 300 locations for the Cu NP (ht) and Cu LO samples on the PI substrate. Cu NP (ht) was selected for the uniformity study as it had a lower *R*_s_ than Cu NP (lt). The results are mapped in Figure 5, where all measured points for each parameter are calculated as the percentage deviation from the average value and presented on a color scale from white (average value) to red (+15%) and blue (−15%). The minimum and maximum values for each parameter are also given with the corresponding percentage deviation in parentheses. Out of the 300 resistance measurement points, the total number of defect lines was 2 for Cu NP and 1 for Cu LO, indicating a high yield of 99.3 and 99.7%, respectively.

The average measured t values for Cu NP (ht) and Cu LO are 113 ± 5 and 40 ± 2 nm, respectively. The thickness map illustrates the effect of the two metal deposition processes on the layer uniformity. The standard deviation for t for both Cu NP (±5%) and Cu LO (±4%) is low, but the overall variation range is larger for Cu NP than for Cu LO. The obtained thickness variation for Cu NP corresponds to the typical values for ROP.^5^ For Cu NP, the variation of the glass slit width of the manually assembled capillary coating unit is one factor contributing to the observed nonuniformity over the printing width (CD). The reduction in Cu NP thickness in MD from the leading edge (top) to the trailing edge (bottom) is likely due to a reduction in the capillary pressure as the ink volume in the open-ended coating unit decreases during the coating process. Layer thickness is influenced not only by capillary coating speed^32^ but, according to our observations, also by the volume of ink dispensed into the coating unit. An automated pressure control of the ink as well as more accurate control of the coater geometry would likely offer improvements in the coating thickness uniformity. For Cu LO, the thickness uniformity follows a radially decreasing profile that is typical for an evaporator with a rotating substrate centered above the evaporation source, where the thickness variation is affected by the distance of the evaporation source, the sample size, and the directionality of the evaporation plume. The thickness variation calculated from the geometry of the vacuum evaporation process for a surface source at 55 cm source distance and 10 cm sample size results in ± 3.2% at the corners of the sample, which is in line with the measured ± 4% for Cu LO.^33^

The average measured *R*_s_ values for Cu NP (ht) and Cu LO are 0.56 ± 0.03 Ω/□ (±5%) and 1.23 ± 0.05 Ω/□ (±4%), respectively. Sheet resistance variation for both Cu NP (ht) and Cu LO largely follows the inverse of thickness variation. The area with slightly lower *t* toward the right edge of the Cu NP (ht) map is more evident as an increase in *R*_s_, as is the effect of decrease in *t* in MD evident as an increase in *R*_s_ in the corresponding locations.

The average measured ρ values for Cu NP (ht) and Cu LO are 6.3 ± 0.4 and 4.9 ± 0.2 μΩ·cm, respectively. The resistivity uniformity for Cu NP (ht) is a product of the printing process uniformity and IPL sintering process uniformity, whereas the resistivity uniformity of Cu LO is mainly determined by the uniformity of the evaporation process. The overall variation in ρ is 6 and 4% for Cu NP and Cu LO, respectively, where the uncertainty in determining the value of *t* from the line profile is the largest factor contributing to the error of each measurement point. These results are comparable to ∼3% variation reported for inkjet-printed and IPL-sintered Ag NP inks, although from a significantly smaller sample number (5) not representing uniformity over a large area.^34^ It should be noted that the values for ρ for the large-area measurements (*L* = 1000 μm, *W* = 50 μm) and basic characterization measurements (*L* = 100 μm, *W* = 16 μm) differed. For Cu NP (ht), the large-area and basic characterization measurements yielded ρ values of 6.3 ± 0.4 and 9.23 ± 0.35 μΩ·cm, respectively. For Cu LO, the ρ values were 4.9 ± 0.2 and 5.33 ± 0.16 μΩ·cm, respectively. The increase in ρ of Cu NP as W decreases may be an indication of significant LER contribution to line resistivity.^35^

**Figure 6:**
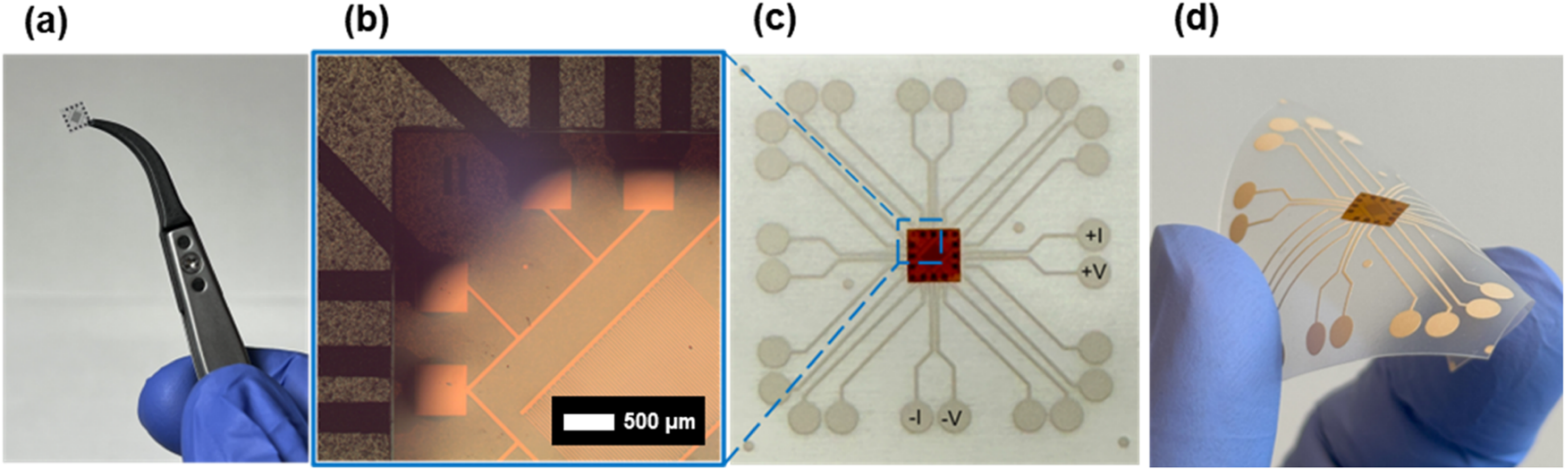
(a) Individual 5 mm × 5 mm flexible IC test chip (Cu LO on the PEN substrate). (b) Optical micrograph of flexible IC test chip edge on PI substrate showing Cu NP (ht) line and contact pad pattern connected to screen-printed Ag electrodes after face-down assembly on a stretchable TPU substrate (c) with a full screen-printed Ag electrode pattern for characterization of the interconnection via contact pads (±I, ±V). (d) Flexible IC test chip from the Cu NP (ht) sample on a stretchable TPU substrate under bending stress.

TPU is a typical stretchable substrate used in wearable electronics that conforms to the body. A preliminary bending test was performed for individual 5 mm × 5 mm flexible IC chips (Figure 6a) assembled on a stretchable TPU substrate (Figure 6b–6d) to test the robustness of the electrical interconnection. In total, each tested sample was subject to 10 bending cycles over three bending radii: 28, 15, and 8 mm. The total resistance over the screen-printed Ag electrode-ICA-flexible ROP chip contact pad and line pattern (*L* = 2425 μm, *W* = 50 μm) remained within 1.4% of the initial measured value for all samples on the PI substrate, with the Cu NP samples suggesting slightly better stability. The promising initial results warrant further studies to investigate the robustness of the flexible ROP conductors and IC to TPU interconnection with, e.g., a higher number of bending cycles and continuous measurement of resistance. The test nevertheless indicates that the interconnection is not strong enough to withstand the stress that the more rigid 125 μm PEN substrate places on the interconnection, as it failed for all PEN samples in the first bending cycle over a bending radius of 8 mm. Mechanical reinforcement of the interconnection using, e.g., adhesives is suggested to improve the robustness.^24,36^ A possible low-cost route to further increasing the thickness and mechanical robustness of the ROP wiring on the chip and to improve the solderability is to use the ROP metal patterning as a seed layer for subsequent electroplating.^37^ The excellent adhesion and cohesion observed for both patterned Cu layers on the PI substrate are promising in light of future mechanical robustness testing (Figure S11). The bending resistance results and description, including additional images of the attached ROP-based flexible test chips, are presented in Figures S12–S14. Finally, an estimate of the contact resistance (*R*_c_) of the Ag electrode to ROP contact pad interconnection was calculated by subtracting the line resistance estimated from the large-area characterization data from the resistance measurement performed in the bending test. The Cu NP samples yielded a lower *R*_c_ value for both full coverage (4.0 Ω) and mesh (5.2 Ω) contact pads compared to Cu LO full coverage and mesh contact pad samples (7.2 and 9.1 Ω, respectively).

## Conclusions

ROP patterning of thin layers at a high resolution offers a notable contribution to improving the sustainability of flexible electronics via a significant reduction in conductive metal volume compared with other existing printing-based methods. Two ROP-based methods for high-resolution, micrometer-level patterning of Cu wiring on flexible substrates (38-μm-thick PI and 125-μm-thick PEN) have been demonstrated: (i) direct patterning of Cu NPs and IPL sintering and (ii) patterning of a polymer resist and an LO process for the vacuum-evaporated Cu. The patterning resolution for Cu LO was higher at the 1–2 μm level due to the low LER of ∼60 nm, whereas the larger LER of the Cu NP process (∼900 nm) limited the current patterning resolution to the 2–4 μm level. It is expected that with further optimization of the NP ink composition and ROP process, the Cu NP process could be improved to the level where the NPs limit the LER and patterning resolution. The results for *t*, *R*_s,_ and ρ over a 10 cm × 10 cm sample area at 300 measurement points show that both processes yield good large-area uniformity: (i) low ρ with comparable uniformity (6.3 ± 0.4 and 4.9 ± 0.2 μΩ·cm for Cu NP and Cu LO, respectively), (ii) high *t* uniformity ≤5% for both methods, and (iii) sufficient *R*_s_ for conductive wiring and electrodes (0.56 ± 0.03 and 1.23 ± 0.05 Ω/□ at 127 and 40 nm thickness for Cu NP and Cu LO, respectively). The ROP LO process was also demonstrated for the patterning of Al, Ag, and Au at a micrometer-level resolution on PI and PEN substrates, exemplifying the potential for using the low-temperature ROP LO process for widening the materials palette from the current available NP inks. Finally, 5 mm × 5 mm test chips with Cu wiring were fabricated and assembled on a stretchable TPU substrate using an electrically conductive adhesive. Preliminary bending resistance results indicated that both ROP-based patterning processes yield a robust electrical interconnection between the flexible chip and TPU on the ultrathin PI substrate, although Cu NPs show potentially better stability in initial bending tests thanks to their higher layer thickness. Comprehensive mechanical testing is planned for future work.

## Supporting Information Available

Summary of key prior work on printed Cu conductors; material usage and cost comparison of ROP Cu NP and ROP LO processes; additional experimental details and images; including photographs; SEM; and optical microscope images; description and results of the bending test and additional images of flexible IC test chips assembled on the TPU substrate (PDF)
