# Supplementary material for: Miniaturized Micrometer-Level Copper Wiring and Electrodes Based on Reverse-Offset Printing for Flexible Circuits
Source: ACS Appl Electron Mater. 2025 Apr 2;7(8):3511–20. doi: 10.1021/acsaelm.5c00230 (PMC12020439; doi:10.1021/acsaelm.5c00230)
Supplement: Supplementary file 1 — el5c00230_si_001.pdf [file el5c00230_si_001.pdf]

SUPPORTING INFORMATION

Miniaturized µm-level copper wiring and electrodes based on reverse-offset printing for flexible circuits

Kim Eiroma†, Asko Sneck†, Olli Halonen†, Tuomas Happonen‡, Henrik Sandberg†, and Jaakko Leppäniemi*†*

† VTT Technical Research Centre of Finland, Ltd., Tietotie 3, FI-02150 Espoo, Finland

‡ VTT Technical Research Centre of Finland, Ltd., Kaitoväylä 1, FI-90590 Oulu, Finland

Corresponding Author

*kim.eiroma@vtt.fi

*jaakko.leppaniemi@vtt.fi

Table S1. Summary of some key prior work on Cu conductors using different printing methods.

Printing method	Ink type	Substrate	Sintering process	$T_{process}$ (°C)	L_{min} (μm)	t (μm)	R_{sq} (Ω/□)	ρ (μΩ·cm)	V_{Cu} in one sq (μm^3)	Ref.
**Screen**	Cu NPs	PET	Forming gas	120	500	5.99	0.098	58.7	1497500	[1]
**Screen**	Cu flakes	PI	In air	170	200	25	0.029	74.6	1000000	[2]
**Screen**	Cu flakes	PI	Hot press	285	201	10.1	0.013	13.13	408050.1	[3]
**Inkjet**	Cu NPs	Glass	Vacuum	325	65	0.75	0.229	17.2	3168.75	[4]
**Inkjet**	CuO NPs	PET	IPL	N/A	80	0.42	0.132	5.54	2688	[5]
**Inkjet**	Cu NPs	PI	Laser	N/A	280	0.27	0.315	8.5	21168	[6]
**EHD**	Cu NPs	Glass	Inert gas	230	30	1	0.092	9.2	900	[7]
**EHD**	Cu NPs	Glass	Vacuum	380	40	3.19	251	80000	5104	[8]
**SIJ**	Cu NPs	Glass	O_2 pump	250	5	1.4	0.058	8.100	35	[9]
**ROP**	Cu NWs	PET/PC	IPL	N/A	7	N/A	31.6	N/A	N/A	[10]
**ROP**	Cu NPs	PC	IPL	N/A	10	0.8	0.23	18.4	80	[11]
**ROP**	Cu NPs	PI	IPL	N/A	2	0.127	0.73	9.23	0.508	Here
**ROP LO**	PVPh + Cu evap.	PI/PEN	IPL	RT	1	0.039	1.37	5.33	0.039	Here

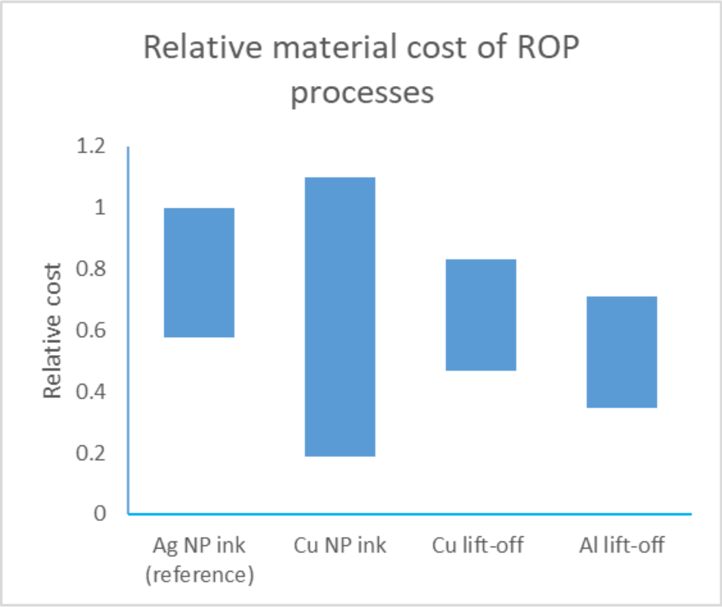

Figure S1. Relative material cost ranges estimated for ROP processes using direct Ag NP ink, direct Cu NP ink, ROP LO of Cu and ROP LO of Al processes that are normalized to Ag ink maximum cost. Total cost is calculated based on available material pricing and usage using 12.5 mm x 12.5 mm sample size and fixed film thickness of 113 nm and 40 nm for the ROP NP and ROP LO processes, respectively. Based on our analysis, the observed range for the cost of the NP ink processes arises from NP ink price/gram, weight percentage of NPs in the ink, and the density of the final sintered metal layer. For the ROP LO process, the range arises from the lift-off solvent usage: solvent price, solvent volume needed for successful LO process, and recycling/re-use of the solvent (i.e. how many samples can be processed with the same solvent bath). As a note, these are simple and qualitative estimates that, at best, can give indication that the two discussed ROP processes are at the same cost-level regarding material usage.

Table S2. Factors affecting the capital and material cost and the environmental impact of the ROP processes shown in Figure S1.

	NP Ag ROP	NP Cu ROP	Cu ROP lift-off	Al ROP lift-off
**Sintering method**	Thermal sintering	Intense pulsed light	No sintering	No sintering
**Generated waste**	Ag NPs	Cu NPs	Cu films + solvent	Al films + solvent
**Lift-off solvent**	No solvent	No solvent	EtOH/MeOH/ACE	EtOH/MeOH/ACE
**Extra tools needed**	Hot plate/oven	Flash sintering system	Evaporator, ultrasonic bath	Evaporator, ultrasonic bath
**Extra tool capital cost**	~1 - 10 k€	~100 - 200 k€	~100 - 200 k€	~100 - 200 k€
**Thermal tolerance needed from substrate**	> 150 °C	> 150 °C	> 50 °C	> 50 °C
**Key factors affecting process cost per sample**	NP ink price/gram	NP ink price/gram	Lift-off solvent price	Lift-off solvent price
	wt% of NPs in ink	wt% of NPs in ink	Solvent consumption	Solvent consumption
	Final sintered layer density	Final sintered layer density	Solvent re-using	Solvent re-using

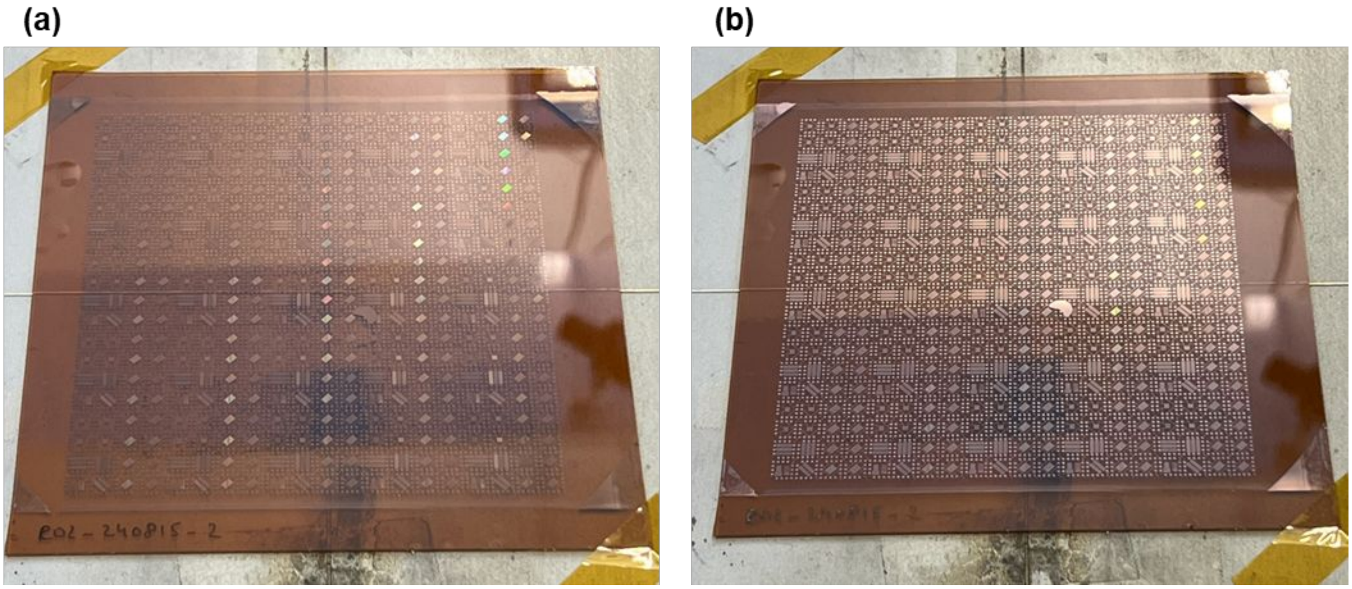

Figure S2. 10x10 cm Cu NP on PI substrate before (a) and after (b) IPL sintering process.

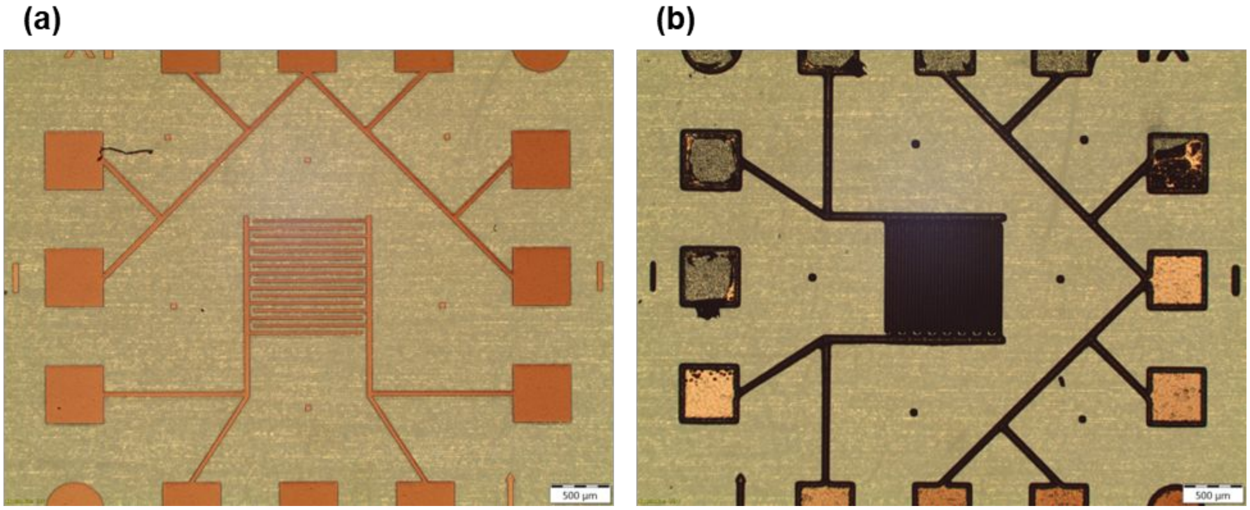

Figure S3. ROP Cu NP patterns on PEN substrate before (a) and after (b) IPL sintering (total pulse energy 344 J).

Table S3. IPL optimization process parameters for Cu NP ink on PI substrate including quantitative resistance (R) and qualitative observations for each pulse voltage (V) and pulse width (t) combination. Total pulse energy (E) was calculated using a formula provided by the equipment manufacturer. Yellow range indicates window of operation where reasonable overall result is obtained. Green indicates the selected optimum parameters for fabricating the ROP Cu NP samples.

Pulse energy E[#]	Voltage V*	Pulse width t**	Resistance R[†]	Layer intact (O) Layer ablated (X)	Adhesion (O/OO/OOO)^
(J)	(V)	(μs)	(Ohm)		
344	2000	1000	X	O	OO
516	2000	1500	4.3	O	O
588	2500	1000	1.3	O	OO
688	2000	2000	1.5	O	OO
881	2500	1500	2.0	O	OOO
1028	2500	1750	1.4	O	OOO
1108	2750	1500	1.3	O	OOO
1138	3000	1250	1.2	O	OOO
1175	2500	2000	1.1	O	OOO
1293	2750	1750	1.4	O	OOO
1365	3000	1500	1.2	O	OOO
1477	2750	2000	1.4	O	OOO
1593	3000	1750	1.4	X	O
1820	3000	2000	X	X	O
[#]*E = [(V/3120)^2.4]t*	**range (V):* 1800-3000	***range (μs):* 100-2000	*† 2-point multimeter* 1.5 mm x 1.5 mm		*^O = low, OO = medium, OOO = high*

Table S4. IPL optimization process parameters for Cu NP ink on PEN substrate including qualitative observations for each pulse voltage (V) and pulse width (t) combination. Total pulse energy (E) was calculated using a formula provided by the equipment manufacturer. No parameter combination was found that provided a low resistance and no melting of the substrate surface and an intact layer. A color change of the patterned Cu layer indicates the onset of sintering, but at this energy level, substrate melting under the patterns is already observed.

**Pulse energy E#**	**Voltage V***	**Pulse width t****	**Conductive (O)† / Not conductive (X)**	**Substrate OK (O) / Substrate melting (X)**	**Layer intact (O) / Layer ablated (X)**	**Cu color change (O) / No color change (X)**
**(J)**	**(V)**	**(μs)**				
228	3000	250	X	O	O	X
258	2000	750	X	X	O	O
267	1800	1000	X	X	O	O
294	1800	1100	X	X	O	O
321	1800	1200	X	XX	O	O
341	3000	375	X	XX	X	O
344	2000	1000	O*	XX	X	O
347	1800	1300	X	XX	X	O
374	1800	1400	X	XX	X	O
401	1800	1500	X	XX	X	O
401	1800	1500	X	XX	X	O
427	1800	1600	X	XX	X	O
454	1800	1700	X	XX	X	O
455	3000	500	X	XX	XX	O
683	3000	750	X	XX	XX	O
# $E = [(V/3120)^{2.4}]t$	*range (V): 1800-3000	**range (μs): 100-2000	† kOhm - Mohm resistance measured			

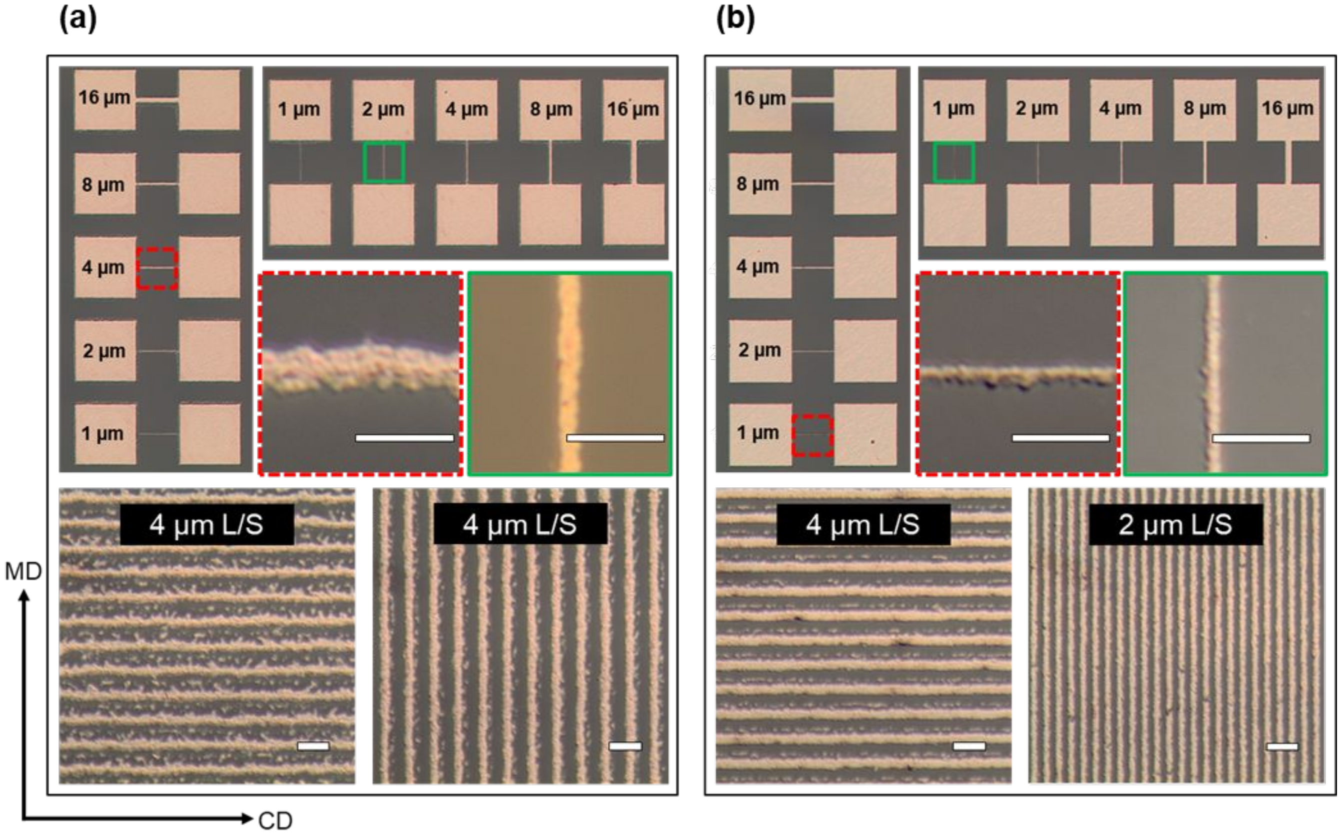

Figure S4. Print quality comparison between direct ROP Cu NP (ht) (a) and Cu NP (lt) (b) on PI substrate. The minimum achieved line-space (L/S) patterning is shown in machine (MD) and cross-machine (CD) directions. The minimum achieved isolated line width is indicated in green (MD) and red (CD). Scale bar is 10 μm.

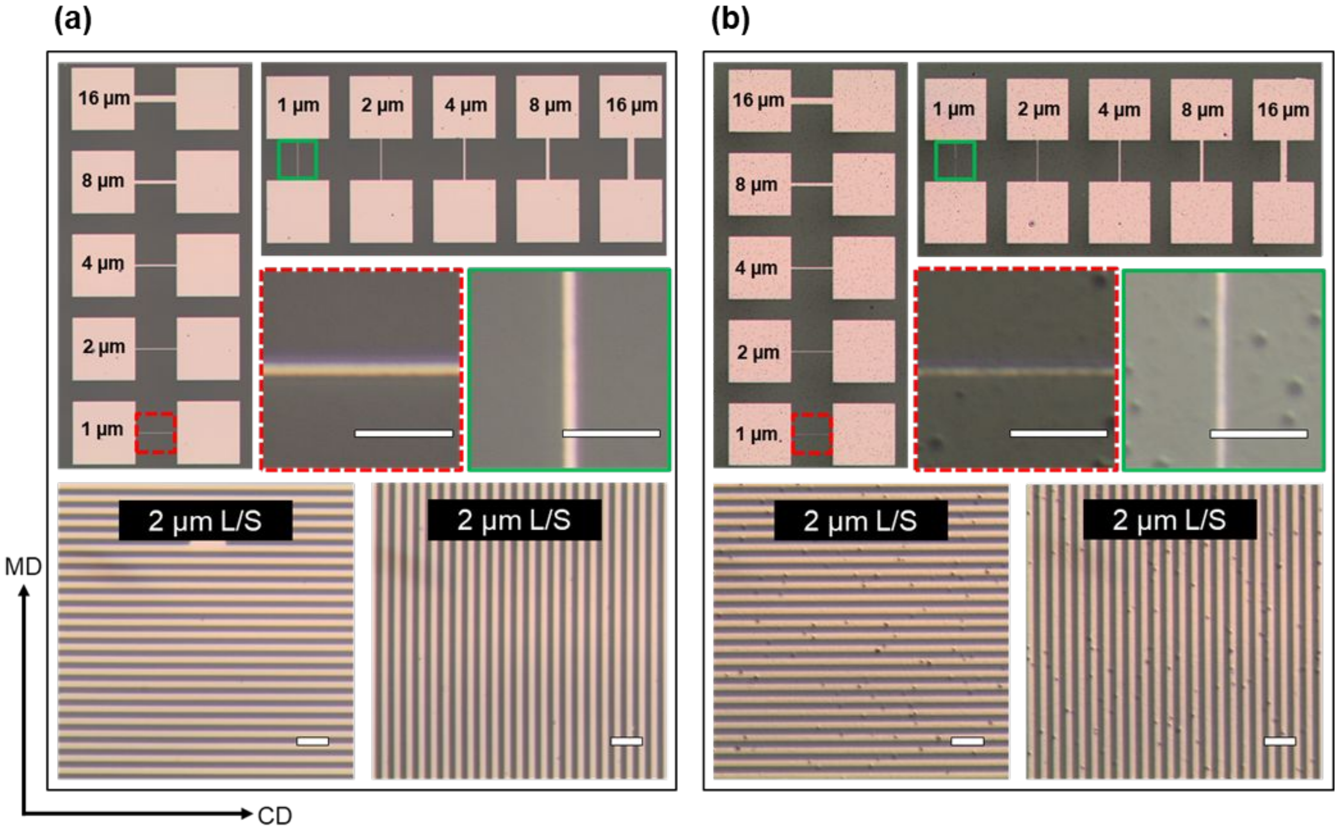

Figure S5. Print quality comparison between ROP lift-off Cu (Cu LO) on PI substrate (a) and PEN substrate (b). The minimum achieved line-space (L/S) patterning is shown in machine (MD) and cross-machine (CD) directions. The minimum achieved isolated line width is indicated in green (MD) and red (CD). Scale bar is 10 µm.

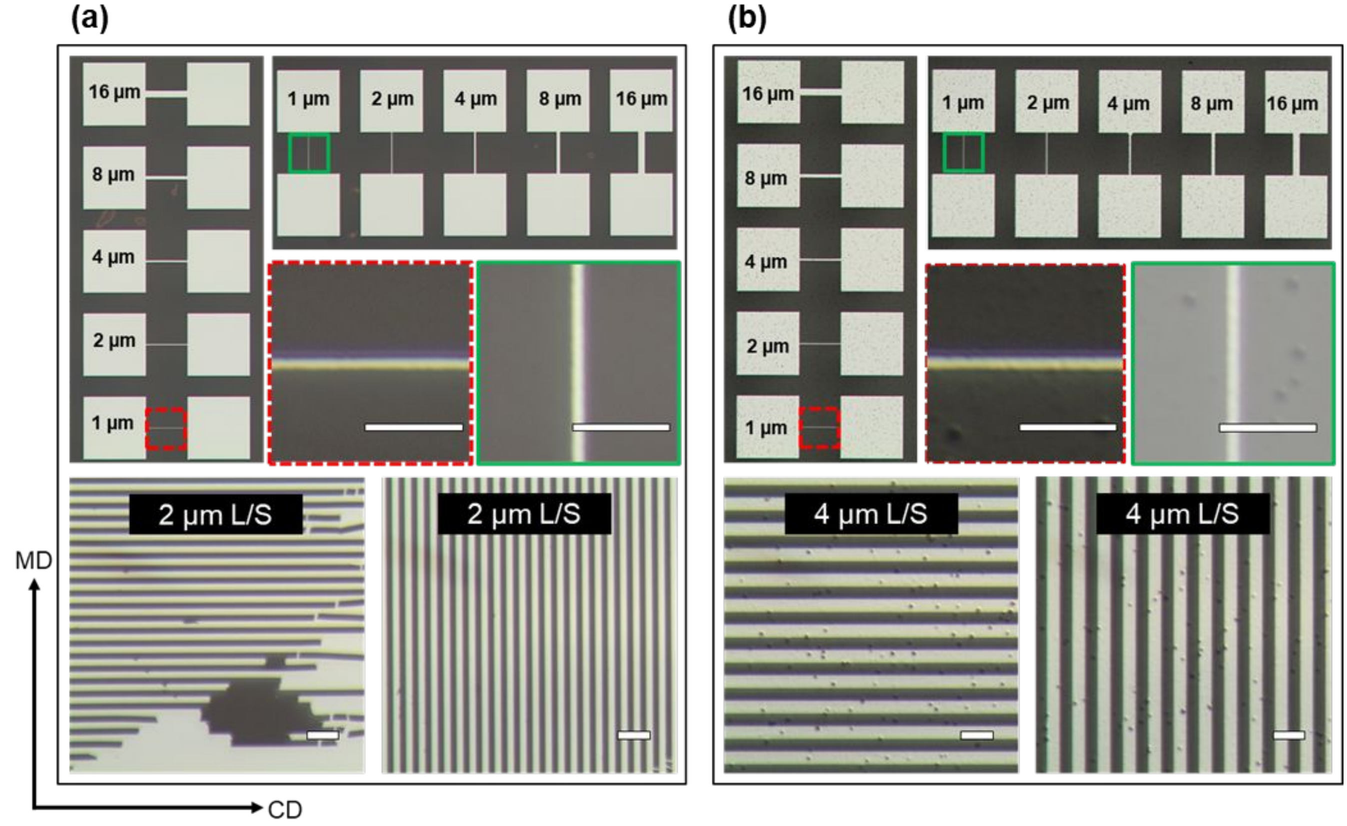

Figure S6. Print quality comparison between ROP lift-off Ag (Ag LO) on PI substrate (a) and PEN substrate (b). The minimum achieved line-space (L/S) patterning is shown in machine (MD) and cross-machine (CD) directions. The minimum achieved isolated line width is indicated in green (MD) and red (CD). Scale bar is 10 µm.

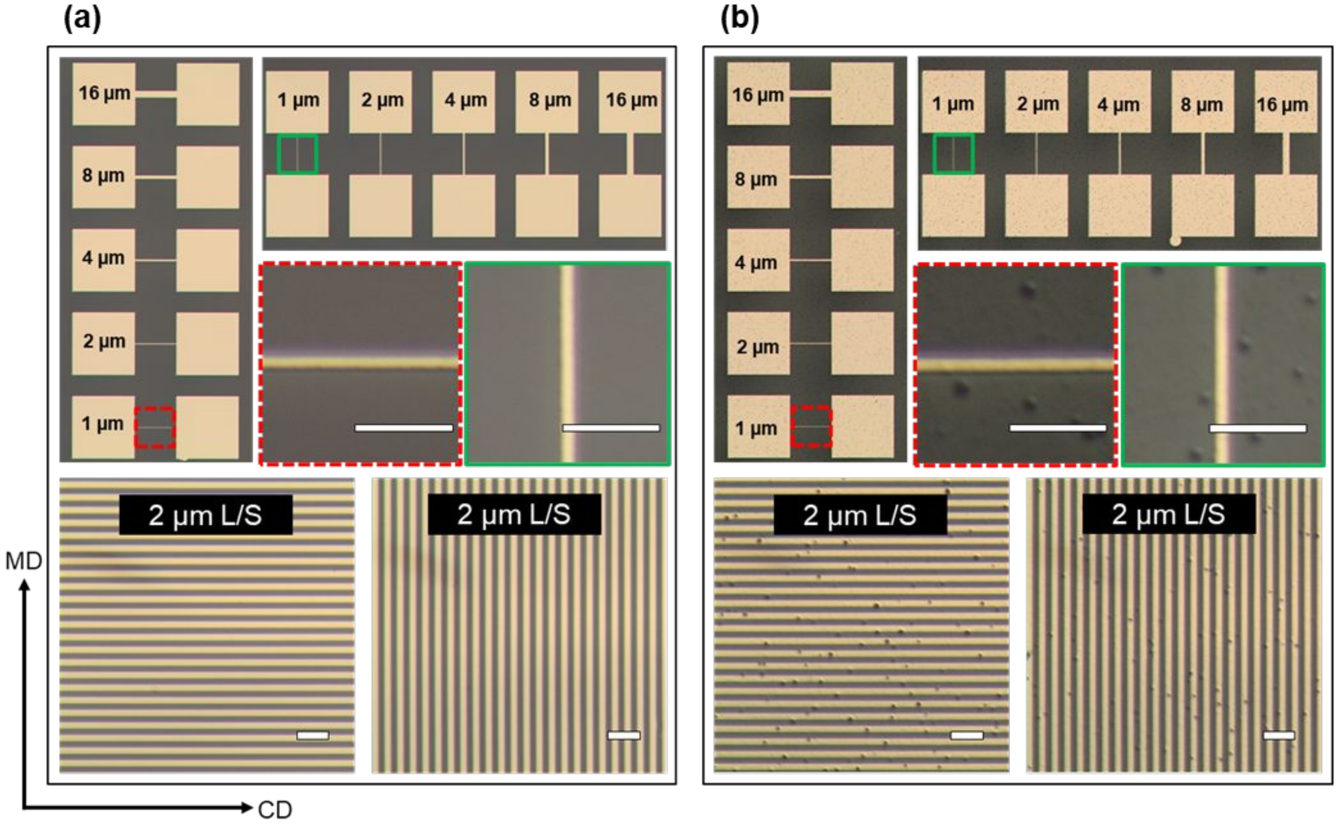

Figure S7. Print quality comparison between ROP lift-off Au (Au LO) on PI substrate (a) and PEN substrate (b). The minimum achieved line-space (L/S) patterning is shown in machine (MD) and cross-machine (CD) directions. The minimum achieved isolated line width is indicated in green (MD) and red (CD). Scale bar is 10 µm.

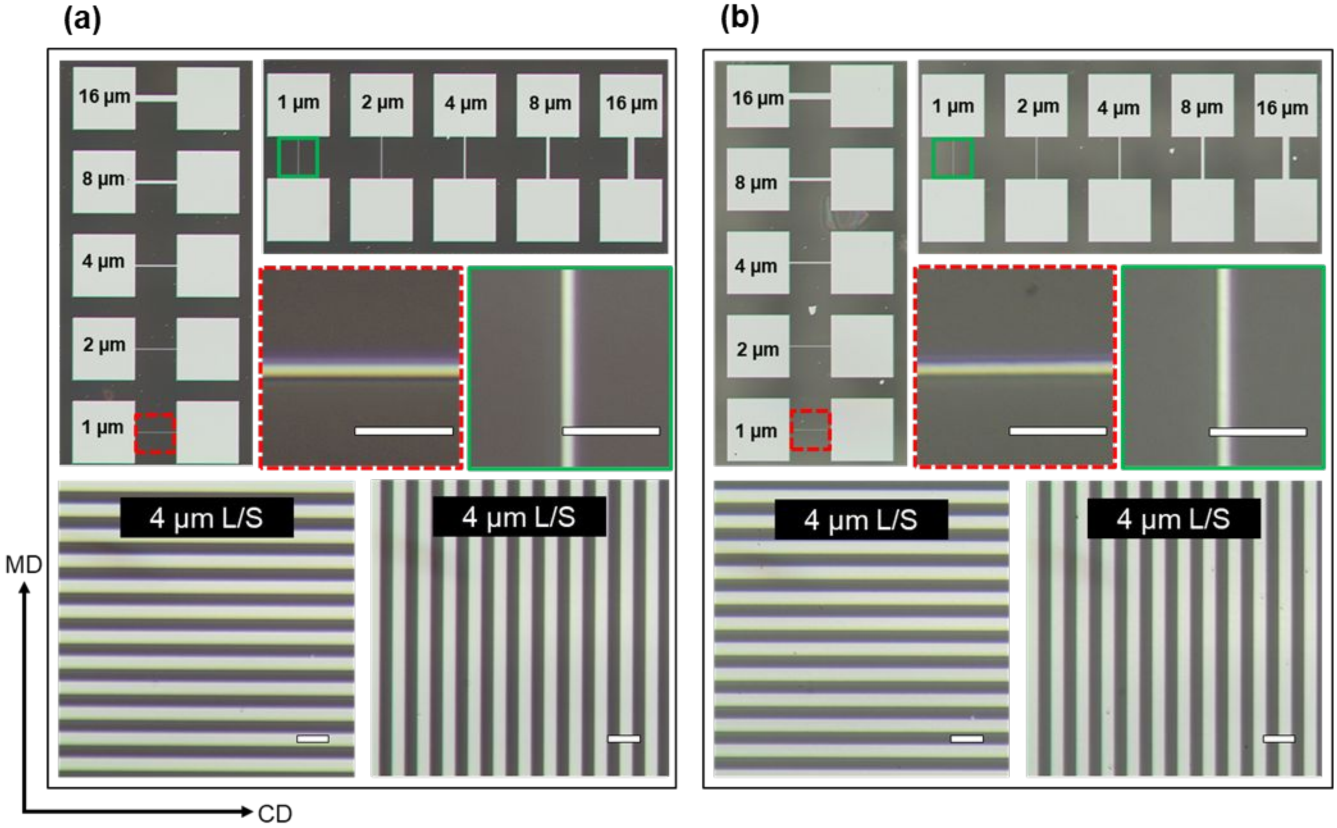

Figure S8. Print quality comparison between ROP lift-off Al (Al LO) on PI substrate (a) and PEN substrate (b). The minimum achieved line-space (L/S) patterning is shown in machine (MD) and cross-machine (CD) directions. The minimum achieved isolated line width is indicated in green (MD) and red (CD). Scale bar is 10 µm.

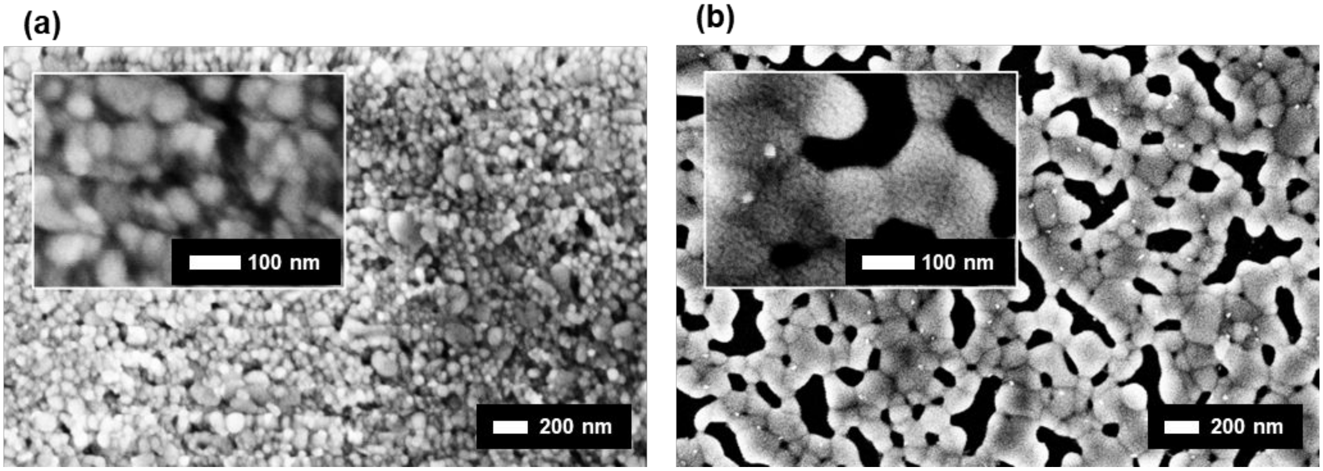

Figure S9. SEM image of Cu NP (lt) morphology before (a) and after (b) IPL sintering. 5 nm of Au has been deposited on the surface of the sample in order to minimize charging of the surface during imaging.

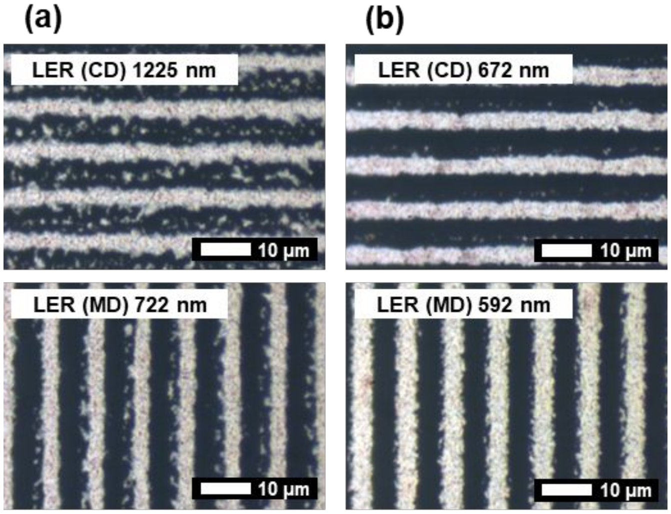

Figure S10. Optical microscope images (100 X) of nominal 4 μm line-space patterns of (a) Cu NP (ht) and (b) Cu NP (lt) on PI substrate in machine (MD) and cross-machine (CD) directions. Line edge roughness (LER) values are indicated for each case.

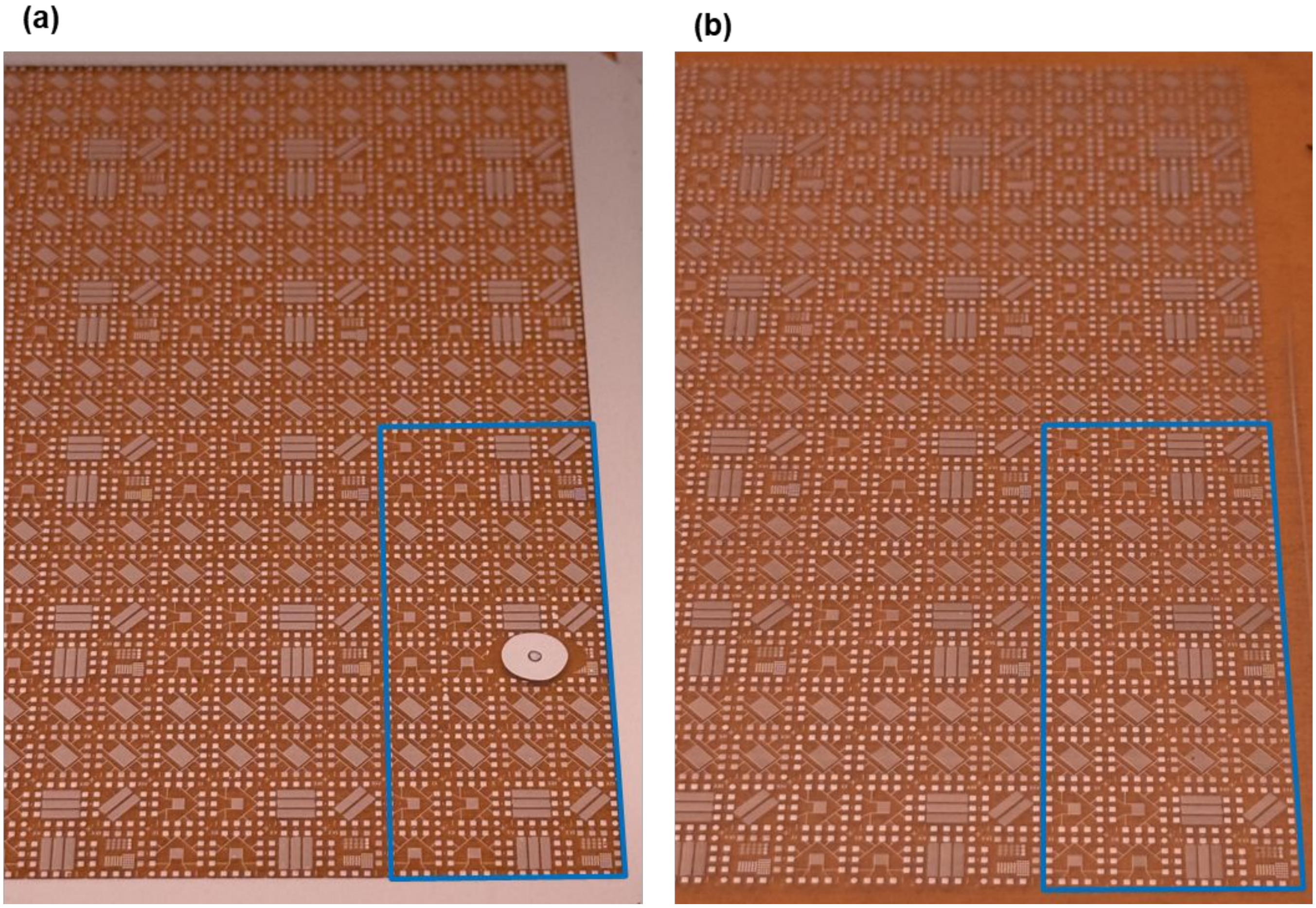

Figure S11. IPL sintered Cu NP (lt) (a) and Cu LO (b) on PI substrate after adhesion tape test. Area within ~2 cm x ~5 cm blue rectangle indicates area tested, showing no signs of poor cohesion of Cu layers or poor adhesion of Cu layers to substrate for both samples.

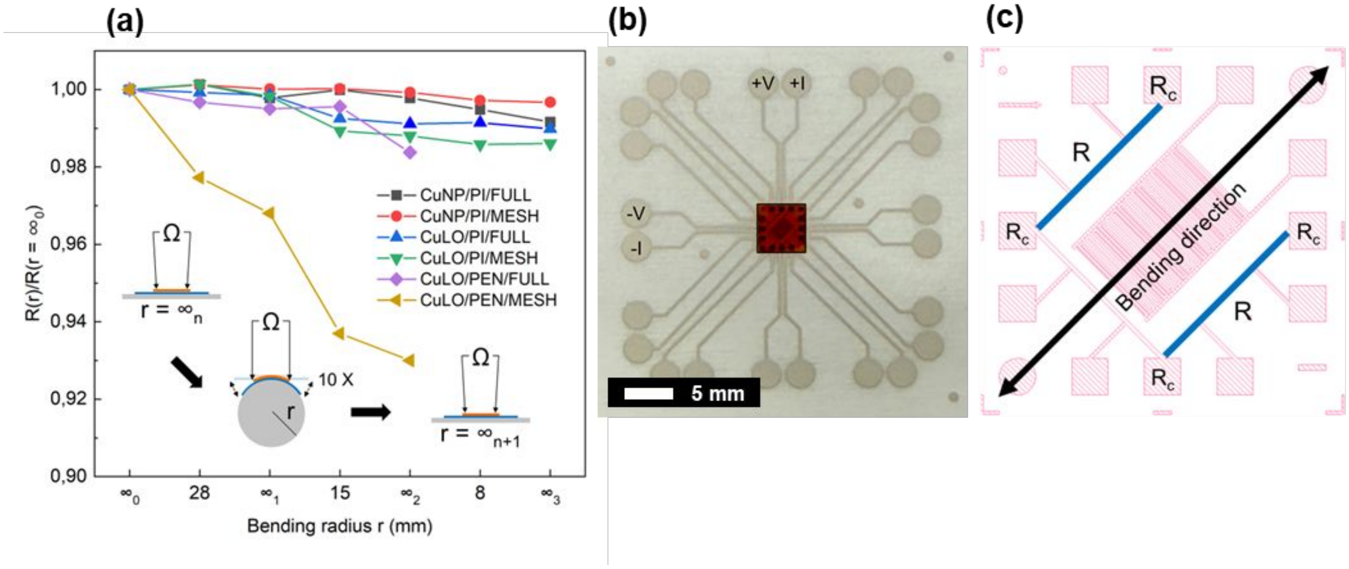

Figure S12. (a) Average measured resistance of a flexible IC test chip sample assembled on stretchable TPU substrate at each bending radius (R(r)) relative to the average initial measured resistance value (R(r = ∞0), plotted against the bending radius (r). (b) Flexible IC chip (Cu NP (ht) / PI) assembled on TPU with ±I and ±V indicating resistance measurement points on screen printed Ag electrodes during bending test. (c) Schematic of 5x5 mm flexible IC test chip layout, where arrow indicates bending direction, R indicates resistance of printed Cu line and Rc indicates contact resistance of Ag electrode / ICA / printed Cu interconnection.

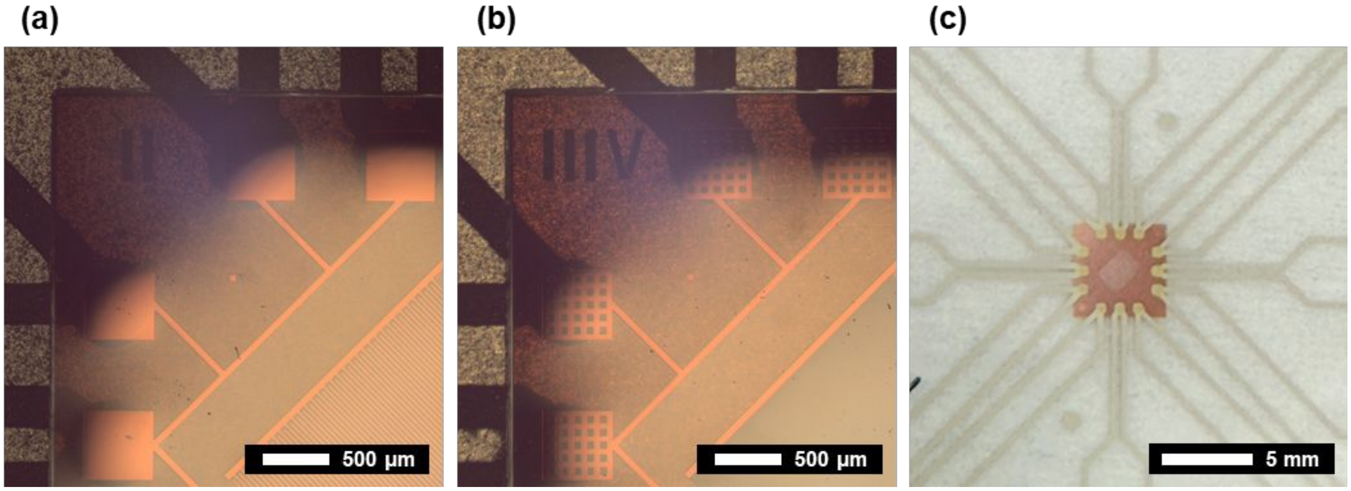

Figure S13. Optical micrograph of flexible IC test chip edge on PI substrate showing Cu NP (ht) line and contact pad pattern connected to screen printed Ag electrodes after face-down assembly on stretchable TPU substrate: (a) full coverage, (b) mesh contact pads. (c) photograph of flexible IC test chip to Ag electrode interconnection from reverse side of TPU substrate.

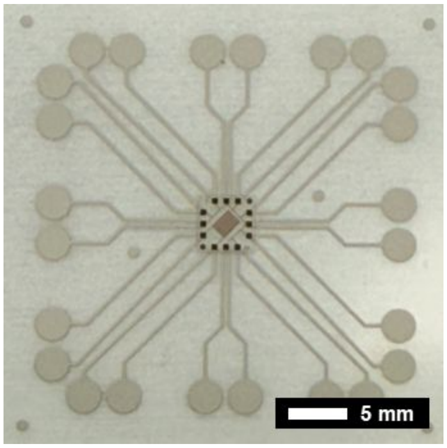

Figure S14. Photograph of flexible IC test chip (Cu LO) on PEN substrate assembled on stretchable TPU substrate with screen printed Ag electrode pattern.

References

[1] S. K. Tam and K. M. Ng, “High-concentration copper nanoparticles synthesis process for screen-printing conductive paste on flexible substrate,” J. Nanoparticle Res., vol. 17, no. 12, pp. 1–12, 2015.

[2] S. Hong, C. Liu, S. Hao, W. Fu, J. Peng, B. Wu, and N. Zheng, “Antioxidant high-conductivity copper paste for low-cost flexible printed electronics,” npj Flex. Electron., vol. 6, no. 1, pp. 1–9, 2022.

[3] C. Vaquero, L. Bilbao, A. Pérez, H. Villaverde, J. Maudes, O. Adarraga, I. Bustero, M. Hayet, J. Caballero, and I. Santamaria, “Silver and copper screen-printed temperature sensors on flexible substrates: The impact of ink sintering conditions and composition,” Appl. Res., no. May, pp. 1–7, 2024.

[4] B. K. Park, D. Kim, S. Jeong, J. Moon, and J. S. Kim, “Direct writing of copper conductive patterns by ink-jet printing,” Thin Solid Films, vol. 515, no. 19 SPEC. ISS., pp. 7706–7711, 2007.

[5] H. Kang, E. Sowade, and R. R. Baumann, “Direct intense pulsed light sintering of inkjet-printed copper oxide layers within six milliseconds,” ACS Appl. Mater. Interfaces, vol. 6, no. 3, pp. 1682–1687, 2014.

[6] J. Niittynen, E. Sowade, H. Kang, R. R. Baumann, and M. Mäntysalo, “Comparison of laser and intense pulsed light sintering (IPL) for inkjet-printed copper nanoparticle layers,” Sci. Rep., vol. 5, pp. 2–11, 2015.

[7] A. Khan, K. Rahman, D. S. Kim, and K. H. Choi, “Direct printing of copper conductive micro-tracks by multi-nozzle electrohydrodynamic inkjet printing process,” J. Mater. Process. Technol., vol. 212, no. 3, pp. 700–706, 2012.

[8] T. T. T. Can, T. C. Nguyen, and W. S. Choi, “High-Viscosity Copper Paste Patterning and Application to Thin-Film Transistors Using Electrohydrodynamic Jet Printing,” Adv. Eng. Mater., vol. 22, no. 3, pp. 1–11, 2020.

[9] N. Shirakawa, K. Murata, Y. Kajihara, K. Nakamura, Y. Kashiwagi, M. Nakamoto, H. Sato, T. Kojima, D. Komiya, K. Shimizu, and K. Masuda, “Fine Pitch Copper Wiring Formed with Super-Inkjet and Oxygen Pump,” Jpn. J. Appl. Phys., vol. 52, p. 05DB19, 2013.

[10] J. Kim, I. Hwang, M. Kim, H. Jung, H. Bae, and Y. Lee, “Simple, Fast, and Scalable Reverse-Offset Printing of Micropatterned Copper Nanowire Electrodes with Sub-10 μm Resolution,” ACS Appl. Mater. Interfaces, vol. 14, no. 4, pp. 5807–5814, 2022.

[11] J. Kim, B. Kang, H. Kim, S. H. Choi, J. Park, H. Jung, Y. Hwang, S. Kwon, K. Woo, and Y. Lee, “High-resolution printing of micrometer-scale copper electrode: From ink formulation and

process optimization to application," J. Mater. Res. Technol., vol. 28, no. September 2023, pp. 131–138, 2024.
